# Transferrin Receptor-Mediated Cellular Uptake of Fluorinated Chlorido[*N*,*N*′-bis(salicylidene)-1,2-phenylenediamine]iron(III) Complexes

**DOI:** 10.1021/acsomega.4c01314

**Published:** 2024-08-05

**Authors:** Astrid Dagmar Bernkop-Schnürch, Martin Hermann, Daniel Leitner, Heribert Talasz, Hubert Aaron Descher, Stephan Hohloch, Ronald Gust, Brigitte Kircher

**Affiliations:** † Department of Pharmaceutical Chemistry, Institute of Pharmacy, CMBI—Center for Molecular Biosciences Innsbruck, CCB—Center for Chemistry and Biomedicine, University of Innsbruck, Innrain 80-82, 6020 Innsbruck, Austria; ‡ Department of Anesthesiology and Critical Care Medicine, Medical University of Innsbruck, Anichstraße 35, 6020 Innsbruck, Austria; § Department of General, Inorganic and Theoretical Chemistry, University of Innsbruck, Innrain 80-82, 6020 Innsbruck, Austria; ∥ Biocenter, Institute of Medical Biochemistry, Protein Core Facility, Medical University of Innsbruck, Innrain 80-82, 6020 Innsbruck, Austria; ⊥ Immunobiology and Stem Cell Laboratory, Department of Internal Medicine V (Hematology and Oncology), Medical University of Innsbruck, Anichstraße 35, 6020 Innsbruck, Austria; # Tyrolean Cancer Research Institute, Innrain 66, 6020 Innsbruck, Austria

## Abstract

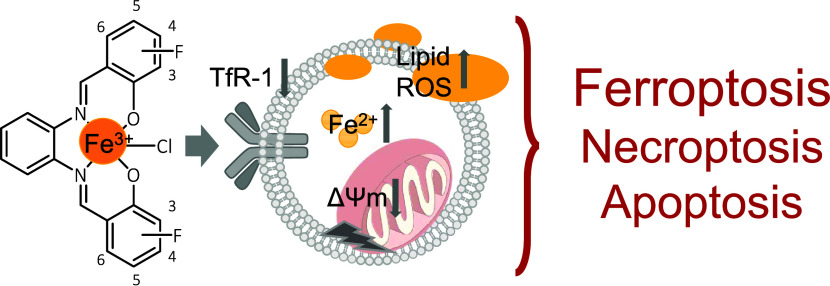

Fluorinated chlorido[salophene]iron(III) complexes (salophene = *N*,*N*′-bis(salicylidene)-1,2-phenylenediamine) are promising anticancer agents. Apoptosis and necrosis induction have already been described as part of their mode of action. However, the involvement of ferroptosis in cell death induction, as confirmed for other chlorido[salophene]iron(III) complexes, has not yet been investigated. Furthermore, the mechanism of cellular uptake of these compounds is unknown. Therefore, the biological activity of the fluorescent chlorido[salophene]iron(III) complexes with a fluorine substituent at positions 3, 4, 5, or 6 at the salicylidene moieties (**C1**–**C4**) was evaluated in malignant and nonmalignant cell lines with focus on the involvement of the transferrin receptor-1 (TfR-1) in cellular uptake, the influence of the complexes on mitochondrial function, and the analysis of the molecular mechanism of cell death. All complexes significantly decreased the metabolic activity in the tested ovarian cancer (A2780, A2780cis), breast cancer (MDA-MB 231), and leukemia (HL-60) cell lines, while the nonmalignant human stroma cell line HS-5 at a concentration of 0.5 μM, which represents the IC_50_ of the complexes in most of the used tumorigenic cell lines, was not affected. The mitochondrial function was impaired, as evidenced by a reduced mitochondrial membrane potential ΔΨm and decreased mitochondrial activity. Besides apoptosis and necroptosis, ferroptosis was identified as part of the mode of action. It was further demonstrated for the first time that fluorinated chlorido[salophene]iron(III) complexes downregulate TfR-1 expression, comparable to ferristatin II, an iron transport inhibitor that acts via TfR-1 degradation. FerroOrange staining further indicated that the complexes strongly increased the intracellular iron(II) level as a driving force to induce ferroptosis. In conclusion, these fluorinated chlorido[salophene]iron(III) complexes are potent, tumor cell-specific chemotherapeutic agents, with the potential to treat various types of cancers.

## Introduction

Platinum complexes derived from the most studied antitumor drug cisplatin^1−3^ are important tools for cancer treatment; however, more recently metal complexes of iron, gold, ruthenium, or gallium have also shown potent anticancer activity.^3−5^ In particular, salophene complexes (salophene = *N*,*N*′-bis(salicylidene)-1,2-phenylenediamine) are of interest, as they are readily accessible from cheap precursors, show catalytic activity, and can form stable complexes with transition metals.^6−11^ The knowledge on the antitumor effect of salophene complexes containing metal ions, such as Ni^2+^, Co^2+^, or Zn^2+^, however, is limited but may be explained by their reduced cytotoxic activity in comparison to their Fe^2+/3+^ counterparts.^12−20^

**Scheme 1:**
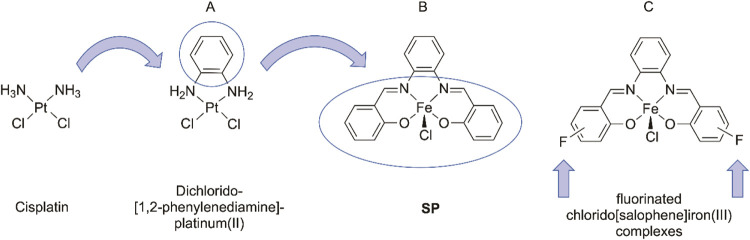
Development of Fluorinated Chlorido[salophene]iron(III) Complexes Starting from Cisplatin

Scheme 1 outlines the design process for the chlorido[salophene]iron(III) complex **SP**. Starting from cisplatin, a 1,2-phenylenediamine component is incorporated instead of the diammine ligands to reduce toxic side effects and to increase tumor selectivity (A in Scheme 1).^18^ The extension of the 1,2-phenylenediamine partial structure to an *N*,*N*′-bis(salicylidene)-1,2-phenylenediamine enables the stable coordination of metal ions. In addition, the formal exchange of the PtCl_2_ substructure for FeCl_3_ led to **SP** (B in Scheme 1) and a shift in the mode of action from coordinative deoxyribonucleic acid (DNA) binding to interference with other intracellular metabolic pathways.^12^ The influence of substituents on the phenylene and/or salicylidene units on antitumor potency and tumor selectivity has been investigated in various structure–activity relationship studies.^21−24^ Especially the introduction of fluorine substituents (C in Scheme 1) resulted in compounds, which were less cytotoxic in the nontumorigenic COS-7 cells and in T cells from healthy individuals.^25^

Although the element fluorine in the form of fluoride minerals is the most frequently occurring halogen, very few naturally occurring organofluorine compounds have been found to date.^26,27^ However, fluorine substituents were used more and more in drug design. Many drugs on the market contain at least one fluorine substituent.^28^ Its incorporation can influence the physicochemical properties, the pharmacokinetic and pharmacodynamic as well as the metabolism and thus the efficacy of drugs.^29,30^

The cytotoxic properties of the chlorido[salophene]iron(III) complexes have been attributed in numerous studies^12,14−16,19,25^ to induction of apoptosis and necrosis, as determined by Annexin V/propidium iodide (PI) staining and/or caspase-3 induction.

Apoptosis has long been considered as the only form of programmed cell death. In the last two decades, however, a large number of new mechanisms have been identified^31−35^ that drive cells to death. These include also ferroptosis and necroptosis.^31−36^ Consequently, the Nomenclature Committee of Cell Death has developed guidelines to categorize the different types into accidental and regulated cell death according to morphology, biochemistry, and function.^37^ Indeed, chlorido[salophene]iron(III) complexes have been shown to induce ferroptosis and necroptosis.^22−24^

Generally, iron(III) ions are receptor-mediated transported into the cells. In the first step, the binding of two ions to apotransferrin converts the protein to transferrin, which is then taken up into the cells by the membrane-bound transferrin receptor-1 (TfR-1) through endocytosis.^38−41^ Interestingly, we have already shown that not only iron(III) but also chlorido[salophene]iron(III) complexes can bind to apotransferrin.^23^ However, to the best of our knowledge, an uptake of chlorido[salophene]iron(III) derivatives by TfR-1 has not been demonstrated yet.

**Scheme 2:**
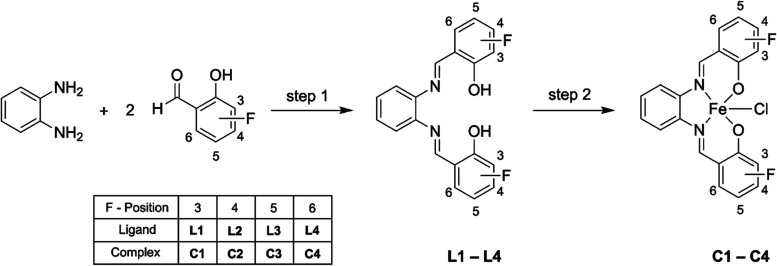
Synthesis of Fluorinated Chlorido[salophene]iron(III) Complexes **C1**–**C4** Step 1: bis-Schiff base ligands (**L1**–**L4**) were synthesized in acetonitrile from 1 equiv of 1,2-phenylenediamine and 2 equiv of 3-, 4-, 5-, or 6-fluorosalicylaldehyde, Step 2: the ligands dissolved in ethanol were reacted with iron(III) chloride to obtain **C1**–**C4**.

Therefore, in this study, we addressed several questions related to the mechanism of action and selected fluorinated chlorido[salophene]iron(III) complexes. We investigated their binding to TfR-1, evaluated their effects on mitochondria, and studied their modes of cell death in detail. Chlorido[salophene]iron(III) complexes **C1**–**C4** with a fluorine substituent at positions 3, 4, 5, or 6 at the salicylidene moieties (Scheme 2) were synthesized and chemically as well as spectroscopically characterized. The biological activity of these compounds was investigated in leukemia (HL-60), breast cancer (MDA-MB 231), cisplatin-sensitive (A2780), and cisplatin-resistant (A2780cis) ovarian carcinoma cell lines as well as in nonmalignant stroma cells (HS-5).

## Results and Discussion

### Chemistry

The synthesis of the fluorinated chlorido[salophene]iron(III) complexes is outlined in Scheme 2 and was performed according to the literature^25^ with some modifications. In brief, bis-Schiff base ligands (**L1**–**L4**) were synthesized from 1 equiv of 1,2-phenylenediamine and 2 equiv of 3-, 4-, 5-, or 6-fluorosalicylaldehyde. The so-formed ligands were then reacted with iron(III) chloride, resulting in the fluorinated chlorido[salophene]iron(III) complexes **C1**–**C4**.

The characterization of the fluorinated ligands and the chlorido[salophene]iron(III) complexes were in line with the literature.^25^ Nuclear magnetic resonance (NMR) and attenuated total reflectance Fourier-transform infrared (ATR-FTIR) spectra can be found in the Supporting Information (Figures S1–S16).

In addition, effective magnetic moments were determined using the Evans method.^42^ The values (see the Experimental Section) ranged from 5.70 to 5.89 μ_B_ for **C1**–**C4**, respectively (Figures S17–S24), and indicated the formation of high spin iron(III) complexes (*S* = 5/2). These results are consistent with those of previously reported iron(III) complexes.^43−45^ As **C1**–**C4** bearing an iron(III) center with an odd number of unpaired electrons, they are detectable by electron paramagnetic resonance (EPR) measurement. These measurements revealed the expected g-values and signature for an iron(III) center (Figure S25).

The electrochemical behavior of the compounds was investigated by cyclic voltammetry (CV), according to a previously described method.^24,45^ The resulting voltammograms of **C1**–**C4** are depicted in Figures S26–S29. The potential (vs ferrocene (Fc)) to reduce **SP** to [salophene]iron(II) amounted to *E*_1/2_ = −728 mV and was shifted upon fluorine substitution to *E*_1/2_ = −624 mV (**C1**), −662 mV (**C2**), −684 mV (**C3**), and −616 mV (**C4**). Fluorine substituents are categorized as electron-withdrawing groups that activate the aromatic ring. The CV data show that they also influence the electron density at the iron(III) center and facilitate the reduction of iron(II). This effect was most pronounced with substituents at positions 3 and 6.

### Biological Evaluation

The biological activity of **C1**–**C4** was investigated in the breast cancer cell line MDA-MB 231, the cisplatin-sensitive and the cisplatin-resistant human ovarian cancer cell lines A2780 and A2780cis as well as in the acute myeloid leukemia cell line HL-60. The complexes were further tested in the nonmalignant human stroma cell line HS-5. **SP** and cisplatin served as references.

#### Concentration-Dependent Reduction of the Metabolic Activity by the Complexes **C1**–**C4**

After the incubation of the respective cells for 72 h with the complexes **C1**–**C4** at concentrations ranging from 0.1 to 10 μM, their viability was determined using a modified 3-(4,5-dimethylthiazol-2-yl)-2,5-diphenyltetrazolium bromide (MTT) assay, which is based on the intracellular conversion of light-yellow colored tetrazolium compounds to orange-colored formazan derivatives, a reaction that only takes place in the presence of functional, active mitochondria.

All complexes reduced the metabolic activity in a concentration-dependent manner (Figures S30–S33). The most active complex was **C4** with IC_50_ values of 0.46, 0.48, and 0.51 μM in MDA-MB 231, HL-60, and A2780cis cells, respectively (Table S1). Only in the case of the A2780 cells, higher concentrations were required to achieve similar effects (IC_50_ = 1.79 μM, Table S1). This cell line, however, was in general less sensitive to the tested compounds.

The complexes reduced the metabolic activity of the cells in the following order: MDA-MB 231 and A2780cis cells: **C4** > **C2** > **C3** > **C1**; A2780 cells: **C1** > **C4** > **C3** > **C2**; HL-60 cells: **C4** > **C2** > **C1** > **C3**. However, it must be mentioned that the cytotoxic effects of the fluorine-substituted chlorido[salophene]iron(III) complexes depended only slightly on the position of the substituent. These results are in line with previously published data on the MDA-MB 231 cell line,^25^ despite using another analysis method.

Since the effects of fluorinated chlorido[salophene]iron(III) complexes on the fibroblastic cell line COS-7 and on healthy human T cells did not unequivocally indicate tumor cell specificity,^25^**C1**–**C4** were further tested on the human nonmalignant stromal cell line HS-5 at concentrations of 0.5 to 10 μM (Figure S34). Due to the limited antimetabolic activity of the compounds on the tumor and leukemia cell lines at a concentration of 0.1 μM (Figures S30–S33), this concentration was omitted for the analysis of HS-5.

Importantly, at concentrations corresponding to the IC_50_ in MDA-MB 231 and A2780cis cells (0.5 μM) none of the complexes reduced the metabolic activity in HS-5 cells. At twice the concentration (1 μM), **C3** completely blocked the metabolic activity, whereas **C4** inhibited it to 56.2%. **C1** and **C2** reduced the metabolic activity solely to 82.7 and 92.2%, respectively. At the highest concentration of 10 μM all complexes reduced the metabolic activity to <10%. Nevertheless, these data indicate tumor-cell specificity of the fluorinated chlorido[salophene]iron(III) complexes at a concentration of 0.5 μM.

Since the antimetabolic activity was similar in all cell lines, the mode of action was determined in more detail only in MDA-MB 231 cells because they are a standard model for investigating interventions in the cellular redox cascade.

#### Cellular Incorporation in MDA-MB 231 Cells Analyzed by Live Confocal Microscopy

We have recently shown that iron(III) complexes with an *N*,*N*′-bis(salicylidene)ethylenediamine scaffold possess fluorescent properties.^45^ Therefore, fluorescence spectra were measured for **C1**–**C4** with the fluorescent dye coumarin 6 as positive control and **SP** as reference. The compounds were dissolved in dimethyl sulfoxide (DMSO) at 5 mM and fluorimetrically evaluated. **SP** showed only low emission, which can be increased by the introduction of fluorine substituents (Figure S35). **C1**–**C4** exhibited a clear emission maximum at 530 nm, which made them suitable for cellular localization studies. Complex **C3** was selected for further studies due to the brightest fluorescence observed by live confocal microscopy.

**Figure 1:**
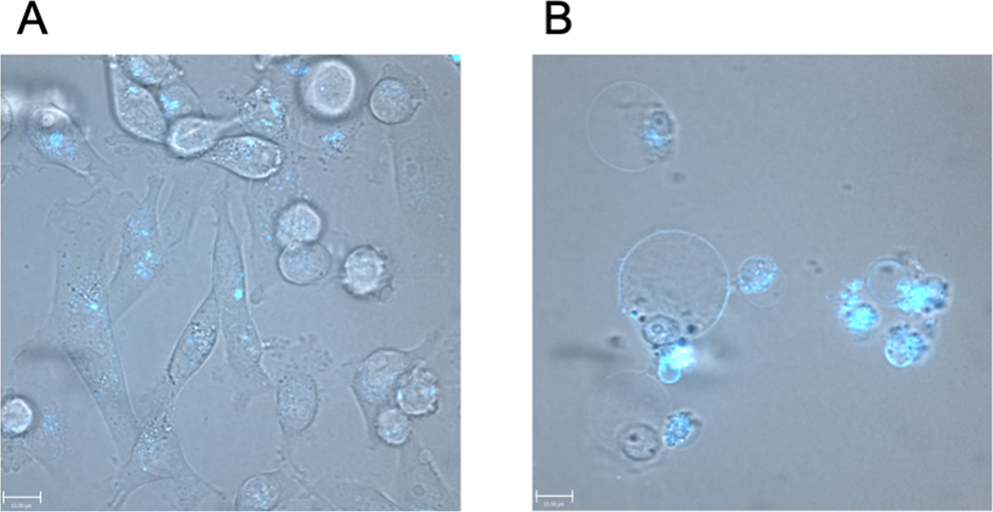
Bright field images of MDA-MB 231 cells before (A) and after treatment for 24 h with **C3** at a concentration of 1 μM (B). Three areas were analyzed per sample. One representative area is shown. Scale bar = 13 μm.

MDA-MB 231 cells were incubated with **C3** at a concentration of 1 μM for 24 h. The cells changed their morphology from the typical spindle-shaped epithelial cells (Figure 1A) to round cells (Figure 1B). Still adherent MDA-MB 231 cells (Figure 1B) clearly show blue fluorescence, indicating the presence of **C3**.

#### Cellular Uptake into MDA-MB 231 Cells Quantified by Graphite Furnace Atomic Absorption Spectrometry (GF-AAS)

**Figure 2:**
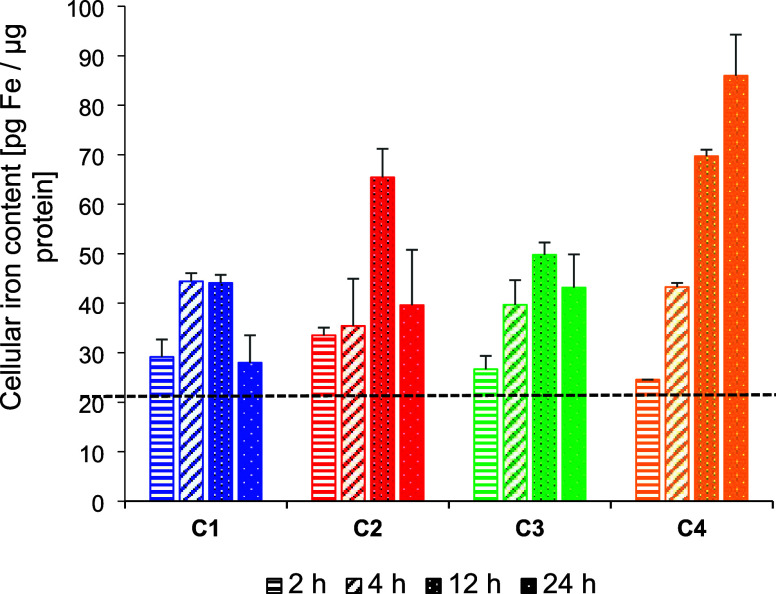
Cellular iron content in MDA-MB 231 cells caused by **C1**–**C4** at a concentration of 1 μM, respectively, measured via GF-AAS after 2, 4, 12, and 24 h of incubation. Iron content of cells incubated without compounds served as reference (dashed line). Data are expressed as mean + standard error (SE) of two independent experiments, measured in triplets.

To quantify the cellular uptake visually determined by live confocal microscopy for **C3**, a cellular uptake study was performed using GF-AAS. Chlorido[salophene]iron(III) complexes are stable in cell culture medium,^11^ and iron can therefore be used as a probe to quantify the cellular uptake by GF-AAS. Thereto, MDA-MB 231 cells were incubated with **C1**–**C4** at a concentration of 1 μM and after various time points (2, 4, 12, and 24 h) the iron content was analyzed. Cells incubated without complexes were used as a negative control to determine the basal iron concentration. This amounts to 21.2 pg Fe/μg protein in MDA-MB 231 cells (Figure 2).

All complexes increased the cellular iron levels in the breast cancer cells, which amounted to 44.7, 65.4, and 49.8 pg Fe/μg protein for **C1**–**C3** after 12 h, respectively. Extending the incubation time to 24 h reduced the values to 28.0, 39.6, and 43.2 pg Fe/μg protein, respectively. In contrast, the amount of iron increased from 69.7 pg Fe/μg protein after 12 h to 86.0 pg Fe/μg protein after 24 h of incubation with **C4**.

To clarify this observation in more detail, we have performed a morphology study with MDA-MB 231 cells incubated for 24 h with **C1**–**C4** at a concentration of 1 μM and analyzed the cells with an inverted fluorescence microscope. MDA-MB 231 cells incubated for 24 h with **C4** revealed a completely different morphology compared to **C1**–**C3** (Figure S36). This could explain the increased iron content after 24 h of incubation with **C4**.

Importantly, the intracellular iron level caused by **C1**–**C4** quantified after 12 h correlates well with the cytotoxic effect determined in the modified MTT assay. The increased iron level after 24 h, however, does not appear to be significant for cytotoxic effects. For example, **C4** had only a slightly higher antimetabolic potency than **C2**, although the iron content was twice as high after 24 h.

#### Analysis of Transferrin Receptor-1 Expression

Transferrin-bound iron(III) is taken up into cells by TfR-1-mediated endocytosis.^38−41^ To test if this pathway is also used by **C1**–**C4**, the complexes were added at concentrations of 0.1 and 1 μM to MDA-MB 231 cells for 12 and 24 h, respectively. TfR-1 expression was analyzed by the JESS automated Western Blot system, which separates protein by size and precisely manages antibody additions, incubations, washes, and even the detection steps, reaching in picogram-level sensitivity.^46,47^ The results are normalized to the amount of protein loaded (β-Actin). To quantify the absolute TfR-1 response to the complexes, we followed the manufacturer’s standard method for 12–230-kDa JESS SM-W004 separation module. FeCl_3_ (1 μM) served as reference.

**Figure 3:**
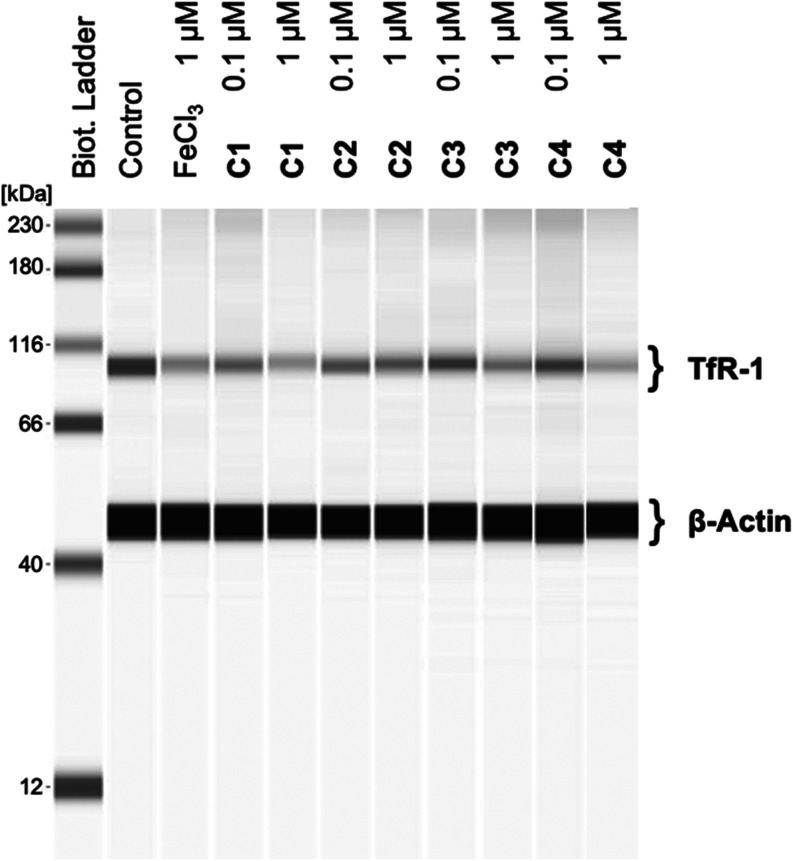
Western blot of the TfR-1 content in MDA-MB 231 cells in the absence (control; lane 2) and the presence of **C1**–**C4** (0.1 and 1 μM; lanes 4–11) for 12 h. FeCl_3_ (1 μM; lane 3) served as reference. The biotinylated molecular weight ladder (Biot. Ladder) is shown as lane 1. β-Actin was used as loading control.

Incubation of the cells with free iron(III) (FeCl_3_) decreased the TfR-1 expression after 12 h to 61.1% (Figure 3 and Table 1) and after 24 h to 50.8% (Figure S37 and Table 1) compared to MDA-MB 231 cells without compound addition (100%).

**Table 1:** TfR-1 Expression (%) in MDA-MB 231 Cells after Treatment with **C1**–**C4** at a Concentration of 0.1 and 1 μM for 12 and 24 h, Respectively, Compared to Untreated Cells

complex	concentration (μM)	TfR-1 expression after 12 h (%)	TfR-1 expression after 24 h (%)
**C1**	0.1	63.0	59.1
	1	53.9	42.9
**C2**	0.1	63.6	50.1
	1	65.5	47.6
**C3**	0.1	61.0	37.0
	1	51.3	41.4
**C4**	0.1	69.2	38.4
	1	51.8	33.7
FeCl_3_	1	61.1	50.8
untreated control		100	100

The complexes reduced the TfR-1 expression after 12 h (Figure 3 and Table 1) as well as after 24 h (Figure S37 and Table 1) already at a concentration of 0.1 μM. **C1**–**C3** (TfR-1 expression: 63.0, 63.6, 61.0%, respectively) reached the effects of FeCl_3_ (1 μM) after 12 h, while **C4** was slightly less active (TfR-1 expression: 69.2%). After 24 h, the TfR-1 expression was reduced to 59.1, 50.1, 37.0, and 38.4%, respectively (Table 1).

When the cells were incubated for 12 h at a concentration of 1 μM, **C1**, **C3**, and **C4** (53.9, 51.3, and 51.8%, respectively) reduced the expression of TfR-1 even more than FeCl_3_ at the same concentration. In the case of **C2**, the effect after 12 h could only marginally be increased when the concentration was raised from 0.1 to 1 μM (63.6 → 65.5%). Extending the incubation time to 24 h increased the influence on TfR-1 in any case. Its expression was reduced at a concentration of 1 μM of **C1**–**C4** to 42.9, 47.6, 41.4, and 33.7%, respectively (Table 1).

This analysis indicates that fluorinated chlorido[salophene]iron(III) complexes induced the same effects on the TfR-1 mediated endocytosis as iron(III) ions. Furthermore, TfR-1 downregulation does not correlate with the accumulation rate of iron in the cells and is therefore not the reason for the reduced iron content in MDA-MB 231 cells treated with **C1**–**C3** at a concentration of 1 μM for 24 h.

#### Analysis of the Iron Uptake in the Presence of a TfR-1 Inhibitor

To further investigate the impact of fluorinated chlorido[salophene]iron(III) complexes on TfR-1 and therefore on the intracellular iron pool, MDA-MB 231 cells were preincubated with ferristatin II for 4 h. Ferristatin II, also known as chlorazol black E (free acid), promotes TfR-1 degradation and thus inhibits iron(III) uptake.^48^

MDA-MB 231 cells incubated for 4 h with ferristatin II at concentrations ranging from 0.5 to 100 μM were analyzed by the JESS automated western blot system^46,47^ and indicated a reduction in TfR-1 expression to 55.2% at 50 μM and to 57.5% at 100 μM (Figure S38 and Table S2). Therefore, further experiments were performed with ferristatin II at 100 μM.

MDA-MB 231 cells were incubated with **C1**–**C4** and stained by FerroOrange. This dye exclusively coordinates the ferrous form of iron,^45^ the intracellular concentration of which is also influenced by transferrin. After endocytotic uptake of transferrin into the cells, bound iron(III) is released in the early endosomes, which is then transported out of the endosome into the cytoplasm. Finally, it is reduced to iron(II). Therefore, visualization of the bivalent iron by the dye FerroOrange allows statements about the labile iron(II) pool.

**Figure 4:**
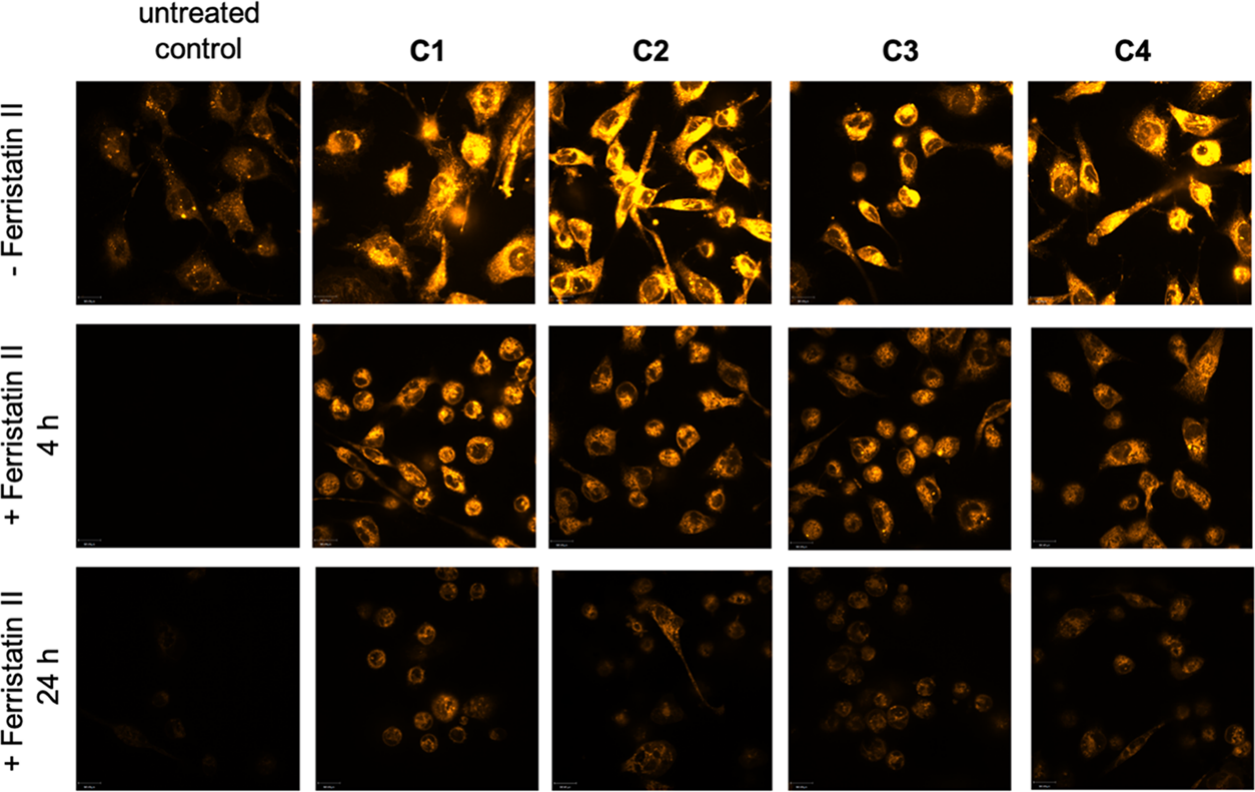
MDA-MB 231 cells imaged by live confocal microscopy after staining with FerroOrange to visualize ferrous iron. Upper row: untreated cells and cells incubated for 4 h with **C1**–**C4** at a concentration of 1 μM; middle row: cells preincubated for 4 h with ferristatin II at a concentration of 100 μM and 4 h with **C1**–**C4**; lower row: cells preincubated for 4 h with ferristatin II at a concentration of 100 μM and concomitantly 20 h with **C1**–**C4**. One representative experiment is shown.

Figure 4 (left column) depicts the physiological amount of ferrous iron in untreated MDA-MB 231 cells. Preincubation with ferristatin II for 4 or 24 h strongly reduced the free available iron(II).

Treatment of the cells with **C1**–**C4** for 4 h drastically increased the labile iron(II) pool, as indicated by the bright yellow color in the cytoplasm (Figure 4, upper row). Pretreatment with ferristatin II for 4 h and subsequent incubation for a further 4 h with the compounds reduced the amount of stainable iron(II) (Figure 4, middle row). After a total incubation time of 24 h (4 h with ferristatin II and subsequent additional incubation with the complexes for 20 h), a clear decrease in dye intensity was observed, confirming a reduced amount of iron(II) in the cells (Figure 4, bottom row).

These results clearly demonstrate that **C1**–**C4** influence the labile iron(II) pool of the cells with the involvement of TfR-1. They caused a high iron(II) level in the cells in a very short time (<4 h). Ferristatin II reduced during 4 h of incubation the TfR-1 expression to about 60%. As a result, fewer receptor molecules are available for the action of **C1**–**C4** during the coincubation, which reduces the iron(II) content in the cytosol compared to the ferristatin II-free cells. Nevertheless, the labile iron(II) pool is still higher than in untreated cells.

#### Mitochondrial Membrane Potential Detected via Live Confocal Microscopy

In order to investigate whether the compounds affect the functionality of mitochondria, MDA-MB 231 cells were incubated exemplarily with **C3** at a concentration of 0.5 μM for 24 h and stained with tetramethylrhodamine methyl ester (TMRM), a cell-permeant dye that accumulates in active mitochondria with intact mitochondrial membrane potential (ΔΨm).

ΔΨm is crucial for maintaining the physiological function of the respiratory chain to generate adenosine triphosphate (ATP) and the transport of charged compounds. The collapse of ΔΨm coincides with the opening of the mitochondrial permeability transition pores, leading to downstream events in the cell death cascade.^49^ As an indication of a disturbed ΔΨm, the accumulation of TMRM is reduced, resulting in a fading of the red color. The cells were counterstained with Hoechst 33342 to image nuclei, and wheat germ agglutinin (WGA) was used to visualize cell morphology.

**Figure 5:**
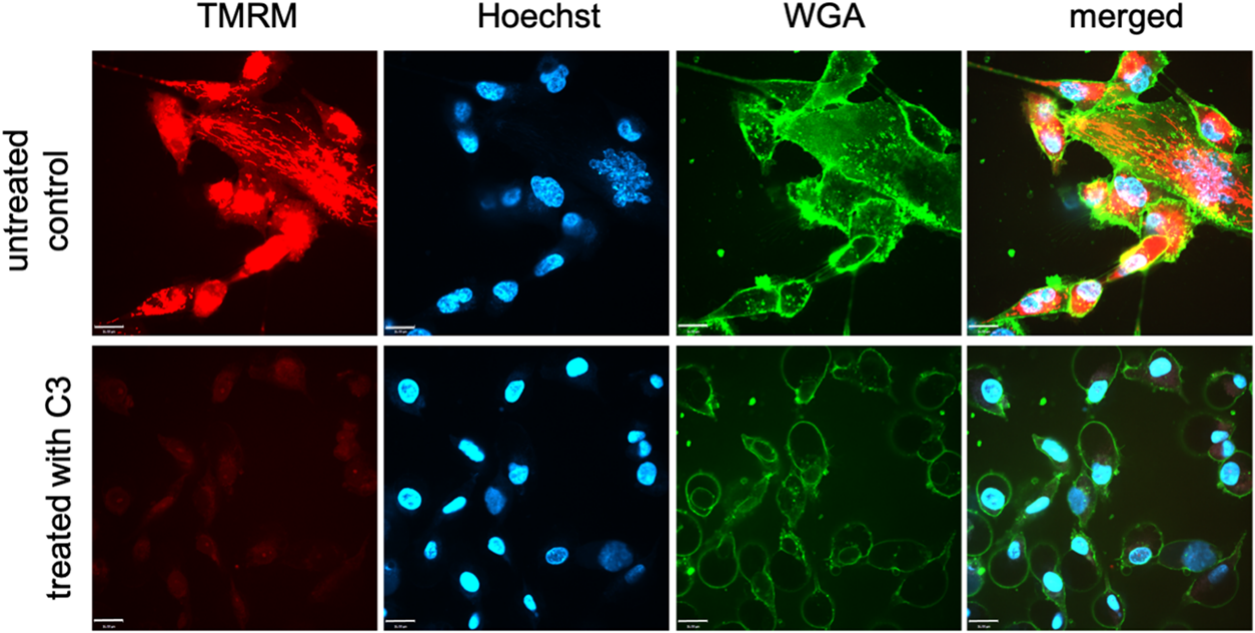
Upper row: untreated MDA-MB 231 cells. Active mitochondria were stained with TMRM (red) and the nuclei with Hoechst 33342 (blue). WGA was used to visualize cell morphology (green). The superimposed images are shown on the right. Lower row: MDA-MB 231 cells treated with **C3** (0.5 μM) for 24 h. Three areas were analyzed per sample. One representative area is shown. Scale bar = 13 μm.

The upper row in Figure 5 shows untreated MDA-MB 231 cells. The typical mitochondrial structure with an intact ΔΨm appears in red, and the cells display the characteristic morphology of spindle-shaped cells. After treatment with **C3**, the accumulation of TMRM was strongly reduced, accompanied by a loss of the epithelial morphology.

#### Photometric Analysis of the Mitochondrial Membrane Potential

These positive results justify a more detailed investigation of the influence of **C1**–**C4** on ΔΨm. Therefore, MDA-MB 231 cells were incubated for 24 h with the complexes at concentrations of 0.1–0.5 μM and were then stained with the cationic dye 5,5,6,6-tetrachloro-1,1,3,3-tetraethylbenzimidazolylcarbocyanine iodide (JC-1). At high mitochondrial concentrations, JC-1 aggregates are formed, which exhibit a red to orange fluorescence and thus display a high ΔΨm. At low mitochondrial concentrations, JC-1 predominantly exists as a monomer, giving rise to green fluorescence. A decrease in the aggregate fluorescence, which can be detected photometrically, is therefore an indication of depolarization (Table 2A).^50^ The mobile ion carrier carbonyl cyanide-*p*-trifluoromethoxyphenylhydrazone (FCCP) served as positive control and was applied at a concentration of 100 μM, as recommended by the manufacturer. FCCP reduced the ΔΨm to 51.2% of the solvent-treated control (Figure 2B), as indicated by the changed intensity of the dye.

**C1**–**C4** concentration-dependently decreased ΔΨm (Table 2B). At a concentration of 0.1 μM, only the complexes **C2** and **C3** changed ΔΨm slightly (ΔΨm = 81.9 and 92.6%), while at the concentrations of 0.25 and 0.5 μM, all complexes were active. At a concentration of 0.5 μM, ΔΨm was reduced to 76.1 (**C1**), 64.5 (**C2**), 70.1 (**C3**), and 57.3% (**C4**), respectively. The effect of **C4** was comparable to that of FCCP at a 200-fold higher concentration.

**Table 2:**
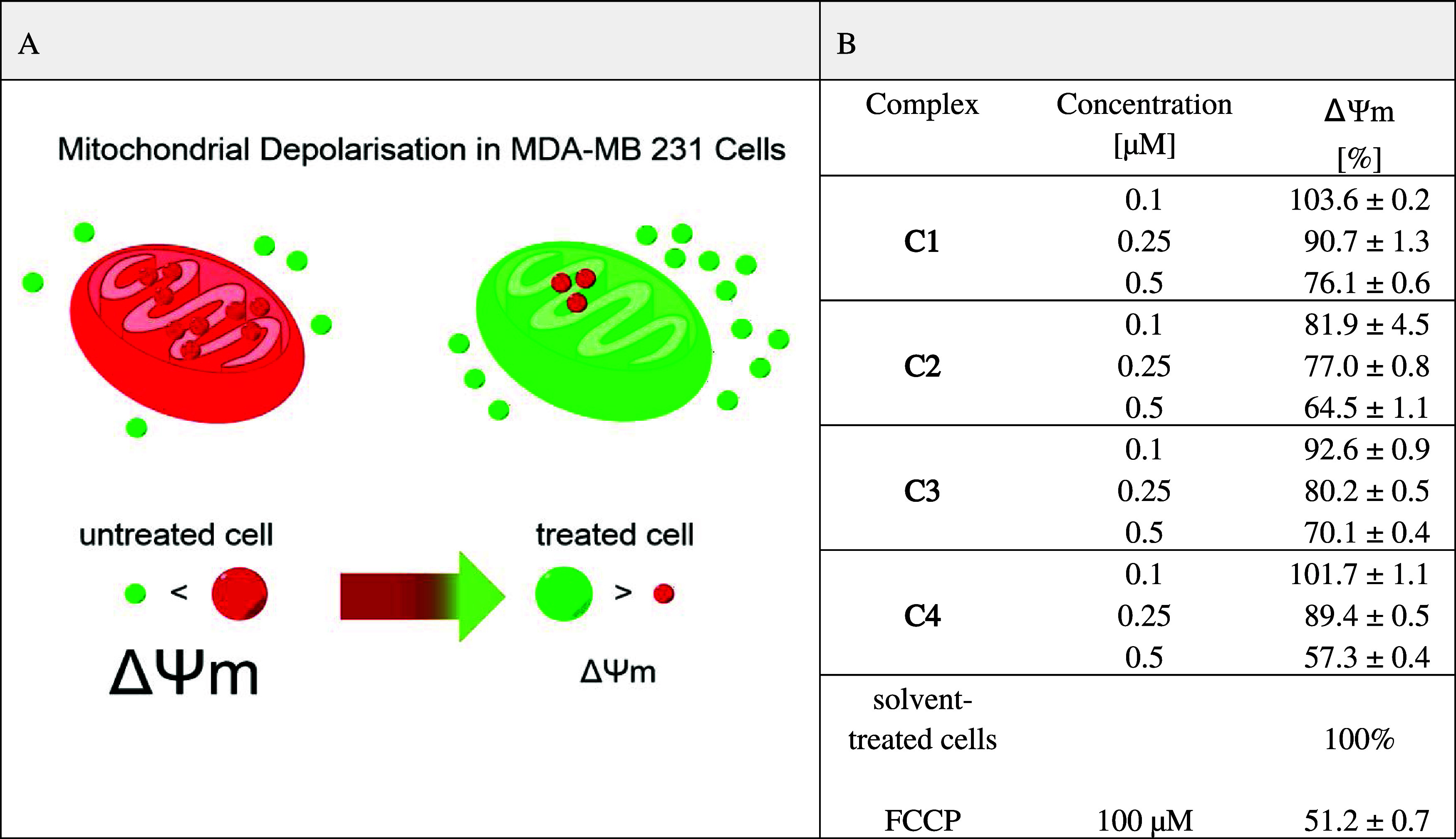
(A) Schematic Representation of the JC-1-based Determination of Mitochondrial Depolarisation in MDA-MB 231 Cells;^a^ (B) ΔΨm of MDA-MB 231 Cells after a 24 h Incubation with **C1**–**C4**^b^

a Red dots represent the JC-1 aggregates and green dots the JC-1 monomers.

b The mean ΔΨm expressed as percentage of the solvent-treated cells (set at 100%) are given in % ± SE of three independent experiments.

These results correlate with the reduced metabolic activity (Figure S30 and Table S1) and the TMRM staining (Figure 5) and underline the pronounced effect of fluorinated chlorido[salophene]iron(III) complexes on mitochondrial activity.

#### Cell Death Analyzed by Live Confocal Microscopy

**Figure 6:**
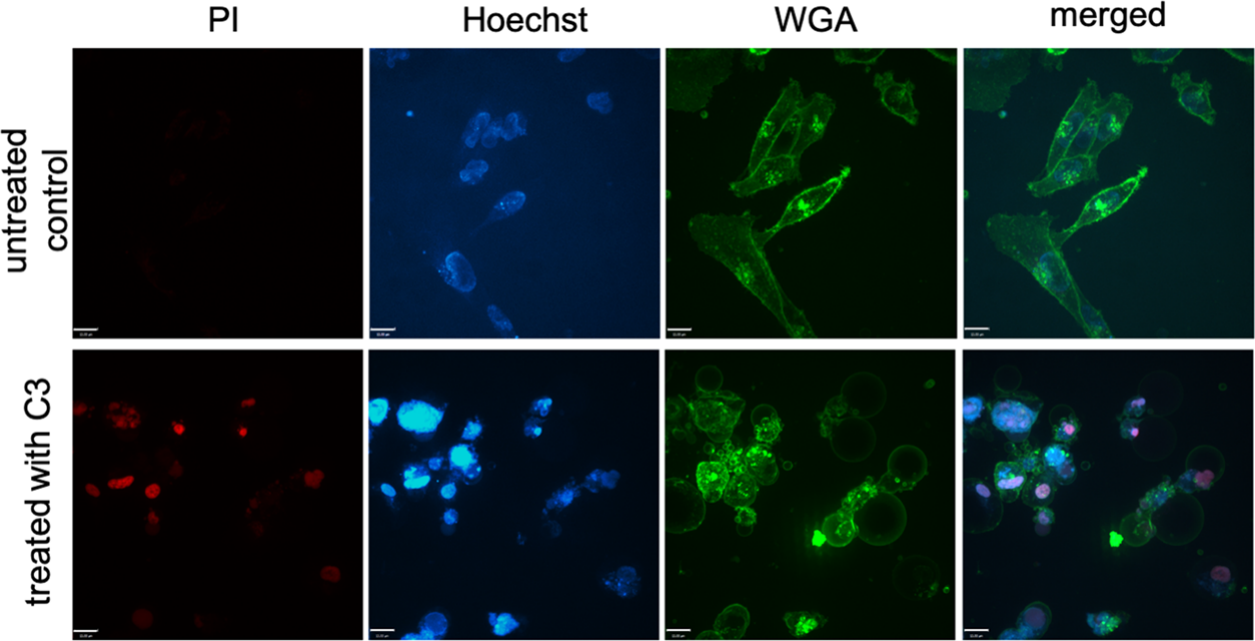
Live confocal images of MDA-MB 231 cells. Upper line: untreated MDA-MB 231 cells. Lower line: cells treated for 24 h with **C3** at a concentration of 0.5 μM. Nuclei of dead cells were colored with PI (red). Hoechst 33342 stains nuclei of cells alive (blue). WGA was used to visualize cell morphology (green). The superimposed images are shown on the right. Three areas were analyzed per sample. One representative experiment is shown. Scale bar = 13 μm.

The reduced ΔΨm mentioned above (Table 2) and the altered cell morphology after treatment with **C3** (Figure 1) strongly indicate cell death. Therefore, MDA-MB 231 cells were stained with PI, a dye, which can only enter dead cells. In addition, the markers Hoechst 33342 and WGA were used as described. In fact, an incubation for 24 h with **C3** at a concentration of 0.5 μM led to cell death. The treated cells showed clear PI staining, which was absent in the cells without the addition of the substance (Figure 6).

#### Cell Death Induction Determined by Flow Cytometry

To further investigate the nature of cell death and to quantify the effects, a flow cytometric analysis was performed using MDA-MB 231 cells incubated with **C1**–**C4** at a concentration of 1 μM for 24 h. Annexin V, which binds to phosphatidylserine located from the inner to the extracellular site of the plasma membrane of apoptotic cells, together with PI staining, makes it possible to distinguish between apoptotic and nonapoptotic cell death.

The flow cytometric results are summarized in Figure S39. A proportion of 69.5% of the untreated cells were alive, 13.5% showed apoptotic cell death, and 15.9% nonapoptotic cell death. The complexes **C1**–**C4** strongly increased the apoptosis rate to 29.5, 16.5, 17.0, and 25.9%, respectively. The majority of cells, however, underwent nonapoptotic cell death after treatment with **C1**–**C4** (39.0, 43.4, 35.1, and 49.8%, respectively), which is consistent with data published by Würtenberger et al.^25^ Furthermore, **C4** with the highest antimetabolic activity (Figure S30), caused the highest amount of nonapoptotic dead cells.

#### Identification of Cell Death through Inhibitor Experiments

The high amount of nonapoptotic cell death caused by **C1**–**C4** raised the question about the kind of cell death induction. It has already been demonstrated that chlorido[salophene]iron(III) complexes induce ferroptosis and necroptosis.^23,24^

Hence, inhibition studies with the ferroptosis inhibitor ferrostatin-1 (Fer-1)^51^ and the necroptosis inhibitor necrostatin-1 (Nec-1)^52^ were performed. If Fer-1 or Nec-1 are able to prevent the inhibition of metabolic activity caused by the complexes, the contribution of ferroptosis or necroptosis to the antitumor effect is very likely. Therefore, MDA-MB 231 and additionally A2780cis cells were incubated for 72 h with **C1**–**C4** (1 μM) in the presence and absence of the respective inhibitor (Fer-1: 1 μM; Nec-1: 20 μM), and metabolic activity was determined as read-out using the modified MTT assay.

Interestingly, both cell lines reacted differently to the use of the inhibitors. In MDA-MB 231 cells, Fer-1 as well as Nec-1 completely prevented the antimetabolic activity of all fluorinated chlorido[salophene]iron(III) complexes (Figure S40). In contrast, a tendency toward ferroptosis as a mode of action was detected for **C2**–**C4** in A2780cis cells, while the results of **C1** indicate a dual mode of cell death (Figure S41).

#### Lipid Peroxidation

Lipid peroxidation is a hallmark of ferroptosis^53,54^ and can be visualized by staining the cells with the dye BODIPY 581/591 C11. The oxidation of the phenylbutadiene moiety of the fluorophore shifts the fluorescence from red to green, which can be quantified by flow cytometry.

MDA-MB 231 and HS-5 cells were treated with **C1**–**C4** at concentrations of 0.1–1 μM for 2 and 4 h, respectively, before the dye was added for 30 min. Concentration-dependent lipid peroxidation was detected just 2 h after addition of **C1**–**C4** (Table 3).

**Table 3:** Percentage of MDA-MB 231 Cells and HS-5 Cells with Lipid Peroxidation after Treatment for 2 and 4 h with Complexes **C1**–**C4** at Concentrations of 0.1, 0.5, and 1 μM, Respectively^a^

cells with lipid peroxidation (%)					
		MDA-MB 231		HS-5	
complex	concentration (μM)	2 h	4 h	2 h	4 h
**C1**	0.1	0.4 ± 0.1*	0.2 ± 0.0	0.5 ± 0.3	0.4 ± 0.2
	0.5	0.9 ± 0.4*	0.9 ± 0.5*	1.1 ± 0.5*	0.5 ± 0.2
	1	1.4 ± 0.9*	1.1 ± 0.4*	0.6 ± 0.2	0.6 ± 0.2
**C2**	0.1	0.2 ± 0.0	0.3 ± 0.1	0.5 ± 0.3	0.5 ± 0.3
	0.5	1.0 ± 0.5*	0.7 ± 0.2*	0.7 ± 0.5**	0.9 ± 0.5*
	1	1.1 ± 0.6*	1.3 ± 0.7*	1.3 ± 0.6	0.5 ± 0.1*
**C3**	0.1	0.4 ± 0.2	0.3 ± 0.1	0.5 ± 0.2*	0.5 ± 0.3
	0.5	0.6 ± 0.0*	0.9 ± 0.2*	0.5 ± 0.3*	0.3 ± 0.1
	1	1.2 ± 0.3*	1.8 ± 1.0*	0.7 ± 0.3	0.4 ± 0.1*
**C4**	0.1	0.5 ± 0.3	0.3 ± 0.2	0.5 ± 0.1**	0.5 ± 0.3
	0.5	1.3 ± 0.6*	2.2 ± 1.0*	0.6 ± 0.2**	0.6 ± 0.2*
	1	1.3 ± 0.1*	3.5 ± 1.9*	0.9 ± 0.4	0.5 ± 0.1
untreated control		0.1 ± 0.0	0.1 ± 0.1	0.1 ± 0.0	0.1 ± 0.0

a Data are expressed as mean ± SE of at least four independent experiments. The asterisks (*p < 0.05 and **p < 0.005 against untreated control) represent statistical significance.

The complexes at a concentration of 1 μM induced in MDA-MB 231 cells after 2 h of incubation significant lipid peroxidation of 1.4 (**C1**), 1.1 (**C2**), 1.2 (**C3**) and 1.3% (**C4**). This effect was increased upon incubation for 4 h in the case of **C2**–**C3** (1.3 (**C2**), 1.8 (**C3**)). The highest lipid peroxidation was detected with the most cytotoxic compound **C4** (3.5%).

HS-5 cells were less sensitive to lipid peroxidation. After 4 h of incubation at a concentration of 1 μM, only 0.6% (**C1**), 0.5 (**C2**), 0.4 (**C3**), and 0.5% (**C4**), respectively, of these cells were tested positive for lipid peroxidation.

## Conclusions

In this study, we extend the knowledge on the anticancer effect of four chlorido[salophene]iron(III) complexes with a fluorine substituent at positions 3, 4, 5, or 6 at the salicylidene moieties (**C1**–**C4**) in various tumor cell lines and the nonmalignant stroma cell line HS-5.

The compounds significantly reduced the metabolic activity of the tumor cells. The complex **C4** was the most active one with an IC_50_ value of about 0.5 μM in MDA-MB 231, HL-60, and A2780cis cells. Only the cisplatin-sensitive cell line A2780 required slightly higher concentrations (IC_50_ = 1.79 μM) to achieve the same effects. At this IC_50_ concentration, the viability of HS-5 cells was not affected.

The antimetabolic activity in MDA-MB 231 cells was caused by a decreased ΔΨm. In addition, the cellular uptake of the intact complexes was confirmed by their specific intrinsic fluorescence using real-time confocal microscopy. The accumulation was then quantified by GF-AAS on the basis of the intracellular iron content. The high cellular uptake after 12 h of all complexes correlated with the antimetabolic activity. The difference between **C1**–**C3** and **C4** after 24 h may be explained by a distinct morphology of the cells.

It is very likely that TfR-1 is involved in the translocation of the fluorinated chlorido[salophene]iron(III) complexes into MDA-MB 231 cells, as they caused a reduced expression of the transporter. Furthermore, the application of the complexes led to increased iron(II) levels in the cells, which was reduced by the coapplication of the TfR-1 inhibitor ferristatin II.

Annexin V/PI staining proved that most of the cells died during a 24 h incubation at a complex concentration of 1 μM. Cell death was due to induction of apoptosis, ferroptosis and/or necroptosis, depending on the cell line used. However, ferroptosis was always involved in the mode of action, as lipid peroxidation, a hallmark of ferroptosis, was induced by all compounds in a concentration-dependent manner.

The fluorine substituents slightly influenced the effects of the chlorido[salophene]iron(III) complexes. Substitution in position 6 of the salicylidene moieties strongly increased the uptake of the resulting complex (**C4**) into the cell and led to a derivative with exceptional in vitro properties. **C1**–**C3** also exhibited high cytotoxicity, although this depended on the cell line used.

In conclusion, these results provide basic in-depth knowledge for the design of novel, highly potent fluorinated chlorido[salophene]iron(III) complexes in the fight against cancer.

## Experimental Section

### General Materials, Methods, and Instrumentation

The chemical reagents and solvents were purchased from commercial suppliers (Sigma-Aldrich (St. Louis, MO), Fisher Scientific (Schwerte, Germany), and Merck (Darmstadt, Germany)) and were used without further purification, if otherwise stated.

#### Analytical Thin-Layer Chromatography

Polygram SIL G/UV254 (Macherey-Nagel, Düren, Germany) plates (0.25 mm layer thickness) with fluorescent indicator and Merck TLC Silica gel 60 F 254 aluminum backed plates. The spots were visualized with UV light (254 nm/365 nm).

#### NMR Spectra of the Ligands

Ultrashield 400 Plus spectrometer (^1^H NMR, 400 MHz; ^13^C NMR, 100 MHz; Bruker, Billerica, MA, USA). The centers of the solvent signal and the tetramethylsilane (TMS) signal were used as internal standards. Deuterated solvents used to measure the NMR spectra were purchased from Eurisotop (Saarbrücken, Germany). Chemical shifts are given in parts per million (ppm). Coupling constants are given in Hertz (Hz).

##### Magnetic Measurements

The complexes in solution were analyzed at a constant temperature of 298.15 K by ^1^H NMR spectroscopy using the Evans method^42^ on an Avance 400 spectrometer operating at 400.14 MHz (Bruker). The measurements of each compound were performed in standard 5 mm NMR tubes containing the paramagnetic samples dissolved in DMSO-*d_6_* with an inert reference of 0.03% TMS against a reference insert tube filled with the same solvent.

##### Attenuated Total Reflectance Fourier-Transform Infrared (ATR-FTIR) Spectroscopy

An α spectrometer (Bruker) was employed. The ATR-FTIR spectra were measured with 32 scans in a wavenumber range covering 4000–400 cm^–1^ and exerting a resolution of 1 cm^–1^. The following abbreviations are used for intensity specifications: w = weak, m = medium strong, s = strong, and br = broad band shape. The wavenumber (ν̅) is given in cm^–1^.

#### High Resolution Mass Spectrometry (HR-MS)

An Orbitrap Elite mass spectrometer (Thermo Fisher Scientific, Waltham, MA) using direct infusion and heated electrospray ionization (HESI) was employed. HR-MS data analysis was carried out with Xcalibur.

#### Elemental Analysis (CHN)

Measurements were performed at the Department of General, Inorganic and Theoretical Chemistry, University of Innsbruck, Austria, with a UNICUBE elemental analyzer from Elementar (Langensbold, Germany).

##### Electron Paramagnetic Resonance (EPR)

The spectra were recorded on a Magnettech M5-5000 X-band EPR spectrometer (Bruker) in a frozen solution of DMSO in 3 mm (outside diameter) fused silica tubes at 98 K. g⊥ (perpendicular) and g|| (parallel) are features arising from an axial type spectrum.

##### Cyclic Voltammetry (CV)

BioLogic SP-150 potentiostat (BioLogic, Seyssinet-Pariset, France) and a three-electrode cell containing a platinum-wire counter-electrode, an Ag/AgCl-electrode with saturated NaCl solution as pseudoreference electrode and a glassy carbon working electrode. Fc (2 mM) served as an internal standard. The supporting electrolyte Bu_4_NPF_6_ (TCI Europe, Zwijndrecht, Belgium) was utilized as received. The software EC-Lab V11.31 was used to evaluate the data.

##### Fluorescence

JC-1 aggregates/monomers were measured with a Tecan Spark Multimode Microplate Reader (Tecan, Grödig, Austria).

##### Absorbance

The Tecan Infinite F50 Plate Reader (Tecan) was used to determine metabolic activity.

##### Graphite Furnace Atomic Absorption Spectrometry (GF-AAS)

M6 Zeeman GFAA-spectrometer (Thermo Fisher Scientific).

#### Real-Time Live Confocal Microscopy

Zeiss Axio Observer Z1 (Zeiss, Oberkochen, Germany) in arrangement with a spinning disc confocal system (UltraVIEW VoX, PerkinElmer, Waltham, MA).

Olympus IX70 inverted fluorescence microscope (Olympus Europe, Hamburg, Germany).

#### Flow cytometry

FACSCanto II (Becton Dickinson, San Jose, CA).

### Chemistry

#### Synthesis and Characterization of the Complexes **C1**–**C4**

##### General Procedure for the Synthesis of the Ligands (Step 1)

One equiv of 1,2-phenylenediamine was dissolved in 8 mL of acetonitrile and heated to reflux. Two equiv of the respectively substituted salicylaldehyde (1.78 mmol) in 10 mL of acetonitrile were added dropwise, and the mixture was refluxed for 8–24 h. Subsequently, the solution was allowed to cool down to room temperature (rt). The precipitated product was collected by filtration and washed with cold acetonitrile to gain **L1**–**L4**. After drying in vacuo, the ligands were obtained as an orange powder. All spectra for the ligands are given as Supporting Information (Figures S1–S12).

##### *N*,*N*′-Bis(3-fluorosalicylidene)-1,2-phenylenediamine (**L1**)

Synthesized from 1 equiv of 1,2-phenylenediamine (100 mg, 0.925 mmol) and 2 equiv of 3-fluorosalicylaldehyde (259 mg, 1.85 mmol). C_20_H_14_F_2_N_2_O_2_; yield: 171 mg (0.49 mmol, 52%), orange solid.

^1^H NMR (400 MHz, DMSO-*d*_6_) δ 13.28 (s, 2H, OH), 9.02 (s, 2H, N=CH), 7.57–7.49 (m, 4H), 7.49–7.36 (m, 4H), 6.96 (ddd, *J* = 7.9, 7.9, 4.6 Hz, 2H).

^13^C NMR (101 MHz, DMSO-*d*_6_) δ 164.20 (d, *J* = 3.1 Hz), 154.34–147.27 (m), 142.10, 128.71, 128.21 (d, *J* = 3.0 Hz), 121.96 (d, *J* = 3.7 Hz), 122.34–117.83 (m), 119.12 (d, *J* = 6.8 Hz).

ATR-FTIR (ν̅) cm^–1^: 1614 s (C=N), 1578 m (C=C), 1401 m (C–N), 1250 s (C–O).

HR-MS (DMSO): *m*/*z* for [M + H]^+^ calcd 353.1196; found 353.1196.

##### *N*,*N*′-Bis(4-fluorosalicylidene)-1,2-phenylenediamine (**L2**)

Synthesized from 1 equiv of 1,2-phenylenediamine (100 mg, 0.925 mmol) and 2 equiv of 4-fluorosalicylaldehyde (259 mg, 1.85 mmol). C_20_H_14_F_2_N_2_O_2_; yield: 187 mg (0.49 mmol, 60%), yellow solid.

^1^H NMR (400 MHz, chloroform-*d*) δ 13.53 (s, 2H, OH), 8.60 (s, 2H, N=CH), 7.35 (ddd, *J* = 7.5, 5.2, 3.7 Hz, 4H), 7.23 (dd, *J* = 5.9, 3.4 Hz, 2H), 6.74 (dd, *J* = 10.7, 2.5 Hz, 2H), 6.64 (ddd, *J* = 8.4, 8.4, 2.5 Hz, 2H).

^13^C NMR (101 MHz, DMSO-*d*_6_) δ 168.97–161.40 (m), 142.16, 135.16 (d, *J* = 11.8 Hz), 128.36, 120.13, 117.14, 107.27 (d, *J* = 22.9 Hz), 104.16 (d, *J* = 23.7 Hz).

ATR-FTIR (ν̅) cm^–1^: 3071 w (arom. C–H), 1613 s (C=N), 1565 s (C=C), 1282 s (C–N), 1191 m (C–O).

HR-MS (DMSO): *m*/*z* for [M + H]^+^ calcd 353.1196; found 353.1196.

##### *N*,*N*′-Bis(5-fluorosalicylidene)-1,2-phenylenediamine (**L3**)

Synthesized from 1 equiv of 1,2-phenylenediamine (100 mg, 0.925 mmol) and 2 equiv of 5-fluorosalicylaldehyde (259 mg, 1.85 mmol). C_20_H_14_F_2_N_2_O_2_; yield: 231 mg (0.66 mmol, 71%), orange solid.

^1^H NMR (400 MHz, DMSO-*d*_6_) δ 12.59 (s, 2H, OH), 8.92 (s, 2H, N=CH), 7.54 (dd, *J* = 9.0, 3.2 Hz, 2H), 7.44 (ddd, *J* = 6.8, 6.3, 3.3 Hz, 4H), 7.29 (ddd, *J* = 8.7, 8.7, 3.2 Hz, 2H), 6.99 (dd, *J* = 9.0, 4.5 Hz, 2H).

^13^C NMR (101 MHz, DMSO-*d*_6_) δ 162.70 (d, *J* = 2.9 Hz), 159.10–152.20 (m), 142.72, 128.55, 120.87 (d, *J* = 23.5 Hz), 122.44–118.64 (m), 118.60 (d, *J* = 7.4 Hz), 117.10 (d, *J* = 23.3 Hz).

ATR-FTIR (ν̅) cm^–1^: 3061 w (arom. C–H), 1614 m (C=N), 1563 s (C=C), 1351 m (C–N), 1269 m (C–O).

HR-MS (DMSO): *m*/*z* for [M + H]^+^ calcd 353.1196; found 353.1201.

##### *N*,*N*′-Bis(6-fluorosalicylidene)-1,2-phenylenediamine (**L4**)

Synthesized from 1 equiv of 1,2-phenylenediamine (100 mg, 0.925 mmol) and 2 equiv of 6-fluorosalicylaldehyde (259 mg, 1.85 mmol). C_20_H_14_F_2_N_2_O_2_; yield: 171.0 mg (0.49 mmol, 52%), orange solid.

^1^H NMR (400 MHz, DMSO-*d*_6_) δ 13.62 (s, 2H, OH), 9.03 (s, 2H, N=CH), 7.57 (dd, *J* = 5.9, 3.4 Hz, 2H), 7.46 (tt, *J* = 7.3, 5.4 Hz, 4H), 6.86–6.75 (m, 4H).

^13^C NMR (101 MHz, DMSO-*d*_6_) δ 166.08–156.53 (m), 142.22, 135.39 (d, *J* = 11.5 Hz), 128.89, 120.64, 113.64 (d, *J* = 3.3 Hz), 108.69 (d, *J* = 12.5 Hz), 105.67 (d, *J* = 20.3 Hz).

ATR-FTIR (ν̅) cm^–1^: 1624 s (C=N), 1585 m (C=C), 1358 m (C–N), 1220 s (C–O).

HR-MS (DMSO): *m*/*z* for [M + H]^+^ calcd 353.1196; found 353.1177.

#### General Procedure of the Synthesis of the Iron(III) Complexes (Step 2)

The respective ligand (1 equiv) was dissolved in 10 mL of ethanol and heated to reflux. One equiv of iron(III) chloride dissolved in 5 mL of ethanol was added, and the reaction mixture was refluxed for 1–2 h. Afterward, the solution was concentrated under reduced pressure. The precipitate was selected and recrystallized from ethanol to yield **C1**–**C4**. The purity was verified by elemental analysis. The ATR-FTIR spectra (Figures S13–S16), Evans ^1^H NMR spectra (Figures S17–S24), the EPR spectra (Figure S25), and cyclic voltammograms (Figures S26–S29) of the complexes are given as Supporting Information.

##### Chlorido[*N,N*′-bis(3-fluorosalicylidene)-1,2-phenylenediamine]iron(III) (**C1**)

Synthesized from 1 equiv of **L1** (100 mg, 0.28 mmol) and 1 equiv of iron(III) chloride (46 mg, 0.28 mmol). Yield: 15.2 mg (0.03 mmol, 12%), black powder.

ATR-FTIR (ν̅) cm^–1^: 1607 s (C=N), 1578 s, 1449 m, 1314 m (C–O).

HR-MS (DMSO): *m*/*z* for [M – Cl] calcd 406.0310; found 406.0293.

Elemental analysis (C_20_H_12_ClF_2_FeN_2_O_2_): calcd C 54.39, H 2.74, N 6.34; found C 54.56, H 3.12, N 6.07.

Magnetic moment (Evans method, DMSO-*d*_6_): μ_eff_ = 5.84 μ_B_.

EPR (9.5 GHz, 98 K): g⊥ = 4.11; g|| = 7.58.

CV: *E*_1/2_ = −624 mV.

##### Chlorido[*N*,*N*′-bis(4-fluorosalicylidene)-1,2-phenylenediamine]iron(III) (**C2**)

Synthesized from 1 equiv of **L2** (100 mg, 0.28 mmol) and 1 equiv of iron(III) chloride (46 mg, 0.28 mmol). Yield: 43 mg (0.097 mmol, 34%), black powder.

ATR-FTIR (ν̅) cm^–1^: 1605 s (C=N), 1581 s (C=C), 1488 m, 1232 m, 1190 m (C–O).

HR-MS (DMSO): *m*/*z* for [M – Cl] calcd 406.0310; found 406.0311.

Elemental analysis (C_20_H_12_ClF_2_FeN_2_O_2_): calcd C 54.39, H 2.74, N 6.34; found: C 54.21, H 3.13, N 6.06.

Magnetic moment (Evans method, DMSO-*d*_6_) μ_eff_ = 5.72 μ_B_.

EPR (9.5 GHz, 98 K): g⊥ = 4.15; g|| = 7.62.

CV: *E*_1/2_ = −662 mV.

##### Chlorido[*N*,*N*′-bis(5-fluorosalicylidene)-1,2-phenylenediamine]iron(III) (**C3**)

Synthesized from 1 equiv of **L3** (100 mg, 0.28 mmol) and 1 equiv of iron(III) chloride (46 mg, 0.28 mmol). Yield: 17 mg (0.038 mmol, 14%), black powder.

ATR-FTIR (ν̅) cm^–1^: 1618 s (C=N), 1533 m, 1372 s (C–N), 1282 m (C–O).

HR-MS (DMSO): *m*/*z* for [M – Cl] calcd 406.0310; found 406.0326.

Elemental analysis (C_20_H_12_ClF_2_FeN_2_O_2_): calcd C 54.39, H 2.74, N 6.34; found: C 54.14, H 3.07, N 6.07.

Magnetic moment (Evans method, DMSO-*d*_6_) μ_eff_ = 5.89 μ_B_.

EPR (9.5 GHz, 98 K): g⊥ = 4.02; g|| = 7.47.

CV: *E*_1/2_ = −684 mV.

##### Chlorido[*N*,*N*′-bis(6-fluorosalicylidene)-1,2-phenylenediamine]iron(III) (**C4**)

Synthesized from 1 equiv of **L4** (100 mg, 0.28 mmol) and 1 equiv of iron(III) chloride (46 mg, 0.28 mmol). Yield: 62 mg (0.14 mmol, 49%), black powder.

ATR-FTIR (ν̅) cm^–1^: 1614 s (C=N), 1532 m, 1370 s (C–N), 1219 m (C–O).

HR-MS (DMSO): *m*/*z* for [M – Cl] calcd 406.0310; found 406.0297.

Elemental analysis (C_20_H_12_ClF_2_FeN_2_O_2_): calcd C 54.39, H 2.74, N 6.34; found: C 54.26, H 2.98, N 6.26.

Magnetic moment (Evans method, DMSO-*d*_6_) μ_eff_ = 5.70 μ_B_.

EPR (9.5 GHz, 98 K): g⊥ = 4.03; g|| = 7.64.

CV: *E*_1/2_ = −616 mV.

### Biological Assays

#### Cell Lines, Reagents, and Complexes

The breast cancer cell line MDA-MB 231 and the acute myeloid leukemia cell line HL-60 were purchased from the German Collection of Microorganisms and Cell Cultures (DSMZ, Braunschweig, Germany). The ovarian carcinoma cell lines A2780 (cisplatin-sensitive) and A2780cis (cisplatin-resistant) were kindly provided by the Department of Gynecology, Medical University Innsbruck. The nonmalignant stroma cell line HS-5 was kindly provided by the Tyrolean Cancer Research Institute. In order to sustain their resistance, A2780cis cells were subjected to biweekly incubation with cisplatin at a concentration of 1 μM. All cell lines were grown in Rosewell Park Memorial Institute (RPMI) 1640 without phenol red (PanBiotech, Aidenbach, Germany), supplemented with a solution of glutamine (2 mM), penicillin (100 U/mL), streptomycin (100 μg/mL), all purchased from Sigma-Aldrich and fetal bovine serum (FBS; 10%; Lonza, Verviers, Belgium) at 37 °C in a 5% CO_2_/95% air atmosphere and fed twice weekly. Cell lines were routinely monitored for mycoplasma infection using the mycoplasma detection kit (MycoStrip, Invivogen, Toulouse, France).

Ferristatin II (Sigma-Aldrich) was dissolved in phosphate-buffered saline (PBS, PanBiotech) and stored at rt. Ferrostatin-1 and necrostatin-1 were purchased from Sigma-Aldrich, and dissolved in DMSO to reach a stock solution of 10 mM, which was stored at −20 °C.

A stock solution of complexes **C1**–**C4** was prepared in DMSO (10 mM) and stored at rt. On the day of the addition of the complexes, the stock solution was diluted with RPMI 1640 without FBS to reach the test concentrations.

#### Analysis of Metabolic Activity

Logarithmically growing MDA-MB 231, A2780, A2780cis, and HS-5 cells were seeded in triplicates in flat-bottomed 96-well plates (Falcon, Corning Life Sciences, Durham, NC) at a density of 1 × 10^4^ cells in 100 μL per well and incubated at 37 °C in a 5% CO_2_/95% air atmosphere for 24 h. Exponentially growing HL-60 cells were seeded also in triplicates into U-bottomed 96-well plates (Falcon) at a density of 2 × 10^4^ cells in 100 μL per well for 2 h. Thereafter, complexes were added to reach the final concentrations in a total volume of 150 μL. After incubation for 72 h, cells were analyzed for metabolic activity using a modified MTT assay (EZ4U kit; Biomedica, Vienna, Austria), according to the manufacturer’s instructions and detection with a Tecan Infinite F50 plate reader. The metabolic activity in the absence of the complexes was set to 100%. The antimetabolic activity of the complexes was calculated in relation to untreated cells.

#### Live Confocal Microscopy

MDA-MB 231 cells (0.2 × 10^6^) were cultured on Ibidi μ-slide 8 well slides (ibiTreat, ibidi, Gräfelfing, Germany) for 24 h before being treated with the complexes for another 24 h. The ferrous iron was detected after 30 min of FerroOrange (final concentration 1 μMol/L; Dojindo, Kumamoto, Japan) staining. The ΔΨm was determined by the addition of TMRM (incubation time 20 min at rt, final concentration 200 nM; (Invitrogen, Thermo Fisher Scientific, Eugene, OR)). Dead cells were stained with PI (final concentration 0.5 μg/mL; Invitrogen Molecular Probes (Thermo Fisher Scientific)). Cells were analyzed in a real-time live confocal microscopy using an inverted microscope in arrangement with a spinning disc confocal system. Cell morphology and nuclei were visualized by adding WGA (final conentration 5 μg/mL) and Hoechst 33342 (final concentration 0.5 μg/mL; Invitrogen Molecular Probes (Thermo Fisher Scientific)). All the images were obtained by using a 40× water immersion objective (Zeiss).

The morphology of the MDA-MB 231 cells after 24 h of incubation with a concentration of 1 μM **C1**–**C4** in comparison to the untreated cells was analyzed with the Olympus IX70 inverted fluorescence microscope (Olympus, Europe).

#### Quantification of Iron by GF-AAS

MDA-MB 231 cells (0.6 × 10^6^) were seeded in 25 cm^2^ flasks (Greiner Bio-One, Kremsmünster, Austria). After reaching 70–80% of confluence (approximately after 24 h), the cell culture medium was replaced by 3 mL of RPMI 1640 supplemented as described above and containing the complexes at a final concentration of 1 μM. The flasks were incubated for 2, 4, 12, and 24 h, respectively. Thereafter, the cells were washed twice with 1 mL of PBS and treated with accutase (Sigma-Aldrich) for 5 min. As soon as all cells detached from the bottom of the flask, 1 mL of RPMI 1640 medium was added, and the mixture was transferred to a 1.5 mL Eppendorf tube and centrifuged at 2300 rcf for 3 min at 4 °C. The cell pellet was washed twice with 1 mL of PBS and stored at −20 °C until analysis. Directly after thawing, the cell pellets were resuspended in 200 μL of Milli-Q water, 0.2% Triton X-100, and lysed by sonication in a cup booster (Sonopuls, Bandelin, Berlin, Germany) three times for 120 s, with cooling at 4 °C, cycle 8, 65% power. The iron content of the cell pellets was determined by GF-AAS using Extended Lifetime Graphite Cuvettes (Thermo Fisher Scientific). Measurements were done at 248.3 and 0.2 nm bandpass under argon atmosphere with Zeeman background correction. The calibration solutions (0.5–12.5 μg/L) were prepared by adequate dilutions of a 1000 mg/L iron standard (TraceCERT, Sigma-Aldrich) stock solution with 0.2% ultrapure nitric acid and Milli-Q water. The graphite furnace temperature program for the determination of iron is shown in Table 4.

**Table 4:** Graphite Furnace Temperature Program for the Determination of Iron in Cell Samples

	phase	temperature (°C)	time (s)	ramp (°C/s)	argon gas flow (L/min)
1	drying	125	40	10	0.2
2	drying	150	10	10	0.2
3	pyrolysis	1100	20	150	0.2
4	atomization	2100	3	0	0.2
5	cleaning	2500	3	0	0.2

The intracellular uptake is presented as the amount of pg Fe/μg protein referred to the cellular protein mass (μg) determined by a classical Bradford assay.

#### Protein Extraction and JESS Automated Western Blot System

MDA-MB 231 cells (2.5 × 10^6^ cells) were seeded in 75 cm^2^ flasks (TPP, Trasadingen, Switzerland) in RPMI 1640 supplemented as described above. For adhesion, the cells were incubated for 24 h and then treated with the respective complex at a concentration of 0.1 or 1 μM for another 24 h. For TfR-1 inhibition, cells were incubated for 4 h at 37 °C with ferristatin II (Sigma-Aldrich). Thereafter, cells were harvested, and each sample was lysed using 30 μL of a modified radio immunoprecipitation assay buffer (containing 50 mM of Tris (pH = 8.0), 150 mM of NaCl, 0.5% NP-40 lysis buffer, 50 mM of NaF, 1 mM of Na_3_PO_4_, 1 mM of phenylmethylsulfonylfluoride (all from Sigma-Aldrich)) and protease inhibitors (ethylenediaminetetraacetic acid (EDTA)-free; Roche, Basel, Switzerland). Total protein concentration was determined by Bradford assay, and samples were run on the JESS automated Western Blot system (ProteinSimple Instruments, Bio-Techne, Minneapolis, MN) according to the manufacturer’s protocol. The following antibodies were used: β-actin and TfR-1 (Cell Signaling Technology, Danvers, MA). The antibodies were diluted with antibody diluents provided by ProteinSimple Instruments (Bio-Techne).

#### Fluorimetric Determination of Mitochondrial Membrane Potential

Analysis of ΔΨm was performed with the JC-1 mitochondrial membrane potential assay kit (Abcam, Cambridge, U.K.) according to the manufacturer’s instructions. The cells were seeded in triplicates at a density of 1.5 × 10^4^ cells per 50 μL and incubated for 24 h with the complexes **C1**–**C4** (0.1, 0.25, or 0.5 μM). The control cells were incubated with FCCP in the dark for 4 h. Thirty minutes before the end of the incubation, 100 μL of JC-1 dye was added to each well. Cells were washed twice with 100 μL of 1× dilution buffer and resuspended in 0.2 mL of assay buffer. The fluorescence of *JC*-aggregates and *JC*-monomers was measured using excitation/emission wavelengths of 535/595 and 485/535 nm, respectively. Analysis was performed on the Tecan Spark Multimode Microplate Reader.

#### Analysis of Cell Death by Flow Cytometry

MDA-MB 231 cells were seeded into flat-bottomed 96-well plates (Falcon) with a density of 1 × 10^5^ cells in 50 μL per well and incubated for 24 h at 37 °C under a 5% CO_2_/95% air atmosphere. The cells were further incubated at 37 °C for 24 h with each compound at a concentration of 1 μM. Thereafter, cells were double-stained in 50 μL of 1× Annexin buffer with 1 μL of Annexin V conjugated to fluorescein isothiocyanate (FITC) dye (green fluorescence, MabTag GmbH, Friesoythe, Germany) and 1 μL of the red fluorescent dye PI (Sigma-Aldrich), which allows discrimination between alive (Annexin V–/PI−), apoptotic (Annexin V+/PI−) and nonapoptotic (Annexin V+/PI+) dead cells. After incubation for 15 min at 4 °C in the dark, flow cytometric analysis was performed on the FACSCanto II (Becton Dickinson, San Jose, CA).

#### Lipid Peroxidation Staining with BODIPY 581/591

MDA-MB 231 and HS-5 cells (1 × 10^5^ each) were seeded into flat-bottom 96-well plates (Falcon) in 100 μL of cell culture medium. After overnight incubation at 37 °C under a 5% CO_2_/95% air atmosphere, the complexes were added at concentrations of 0.1, 0.5, and 1 μM. After 2 and 4 h, cells were detached with accutase and centrifuged at 200 rcf for 5 min. Meanwhile, a 2.5 μM BODIPY 581/591 staining solution (Invitrogen, Thermo Fisher Scientific) was prepared in PBS, and the cells were resuspended in 100 μL of the staining solution and incubated for 30 min at 37 °C in the dark. After centrifugation for 10 min at 200 rcf and 4 °C, the pellet was resuspended in 200 μL of PBS and immediately analyzed by flow cytometry on the FACSCanto II.

### Statistical Analysis

The Mann–Whitney *U* test was used to analyze the metabolic activity in the absence and the presence of a variable concentration of the test compounds (NCSS software, Kaysville, UT).

IC_50_ values were calculated with Quest Graph IC_50_ Calculator from AAT Bioquest, Inc. (Pleasanton, CA).

## Supporting Information Available

^1^H NMR, ^13^C NMR, and ATR-FTIR spectra of all ligands **L1**–**L4**; ATR-FTIR spectra; Evans ^1^H NMR spectra; EPR spectra and cyclic voltammograms of **C1**–**C4**; determination of metabolic activity, fluorescence measurement, inverted fluorescence microscopy, western blot analysis, and flow cytometry; determination of the metabolic activity with inhibitors (PDF)
Molecular formula strings of **C1**–**C4** with IC_50_ ± SE values for MDA-MB 231, HL-60, A2780, and A2780cis (CSV)

## Abbreviations

ATP: adenosine triphosphate
ATR-FTIR: attenuated total reflectance Fourier-transform infrared
biot: biotinylated
CV: cyclic voltammetry
DMSO: dimethyl sulfoxide
DNA: deoxyribonucleic acid
EPR: electron paramagnetic resonance
equiv: equivalent
FBS: fetal bovine serum
Fc: ferrocene
FCCP: cyanide-*p*-trifluoromethoxyphenylhydrazone
Fer-1: ferrostatin-1
FITC: fluorescein isothiocyanate
GF-AAS: graphite furnace atomic absorption spectrometry
HESI: heated electrospray ionization
HR-MS: high-resolution mass spectrometry
JC-1: 5,5,6,6-tetrachloro-1,1,3,3-tetraethylbenzimidazolylcarbocyanine iodide
ΔΨm: mitochondrial membrane potential
MTT: 3-(4,5-dimethylthiazol-2-yl)-2,5-diphenyltetrazolium bromide
Nec-1: necrostatin-1
NMR: nuclear magnetic resonance
PBS: phosphate-buffered saline
PI: propidium iodide
RPMI: Rosewell Park Memorial Institute
rt: room temperature
salophene: **SP**, *N*,*N*′-bis(salicylidene)-1,2-phenylenediamine
SE: standard error
TfR-1: transferrin receptor-1
TMRM: tetramethylrhodamine methyl ester
TMS: tetramethylsilane
WGA: wheat germ agglutinin
