# Supplementary material for: Transferrin Receptor-Mediated Cellular Uptake of Fluorinated Chlorido[N,N′-bis(salicylidene)-1,2-phenylenediamine]iron(III) Complexes
Source: ACS Omega. 2024 Aug 5;9(33):35394–407. doi: 10.1021/acsomega.4c01314 (PMC11340086; doi:10.1021/acsomega.4c01314)
Supplement: Supplementary file 1 — ao4c01314_si_001.pdf [file ao4c01314_si_001.pdf]

Supporting Information

Transferrin Receptor-Mediated Cellular Uptake of Fluorinated Chlorido[N,N′-bis(salicylidene)-1,2-phenylenediamine]iron(III) Complexes

*Astrid Dagmar Bernkop-Schnürch[1], Martin Hermann[2], Daniel Leitner[3], Heribert Talasz[4], Hubert Aaron Descher[1], Stephan Hohloch[3], Ronald Gust[1], Brigitte Kircher[*5,6]*

[1]Department of Pharmaceutical Chemistry, Institute of Pharmacy, CMBI—Center for Molecular Biosciences Innsbruck, CCB—Center for Chemistry and Biomedicine, University of Innsbruck, Innrain 80-82, 6020 Innsbruck, Austria
[2]Department of Anesthesiology and Critical Care Medicine, Medical University of Innsbruck, Anichstraße 35, 6020 Innsbruck, Austria
[3]Department of General, Inorganic and Theoretical Chemistry, University of Innsbruck, Innrain 80-82, 6020 Innsbruck, Austria
[4]Biocenter, Institute of Medical Biochemistry, Protein Core Facility, Medical University of Innsbruck, Innrain 80-82, 6020 Innsbruck, Austria
[5]Immunobiology and Stem Cell Laboratory, Department of Internal Medicine V (Hematology and Oncology), Medical University of Innsbruck, Anichstraße 35, 6020 Innsbruck, Austria
[6]Tyrolean Cancer Research Institute, Innrain 66, 6020 Innsbruck, Austria

*Correspondence: brigitte.kircher@i-med.ac.at

Content

Chemistry S3

^{1}H-NMR spectra of ligands S3

^{13}C-NMR spectra of ligands S5

ATR-FTIR spectra of ligands and complexes S7

Evans ^{1}H-NMR spectra of complexes S11

EPR spectra of complexes S15

Cyclic voltammetry of complexes S16

Biological investigations S18

Determination of the metabolic activity S18

Fluorescence measurement S21

Inverted fluorescence microscopy S21

Western Blot analysis S22

Flow cytometry S24

Determination of the metabolic activity with inhibitors S25

Chemistry

^{1}H-NMR spectra of ligands

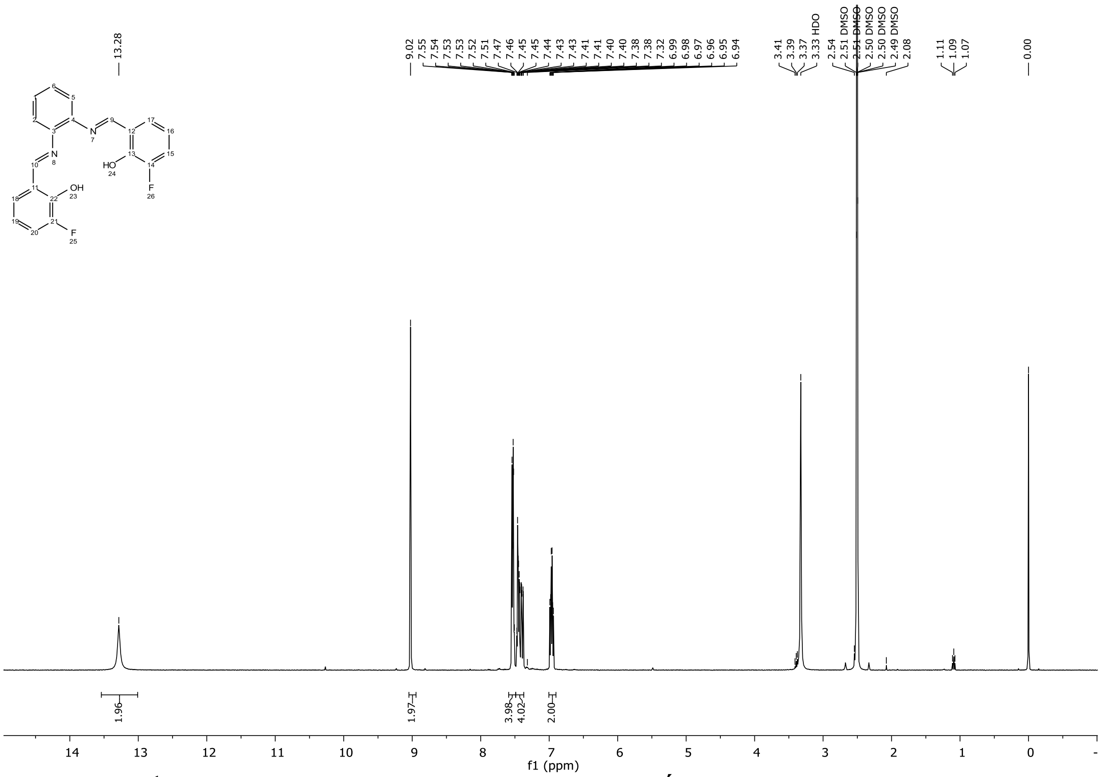

Figure S1: ^{1}H-NMR (400 MHz, DMSO-d_6) of *N,N´*-bis(3-fluorosalicylidene)-1,2-phenylene-diamine (**L1**)

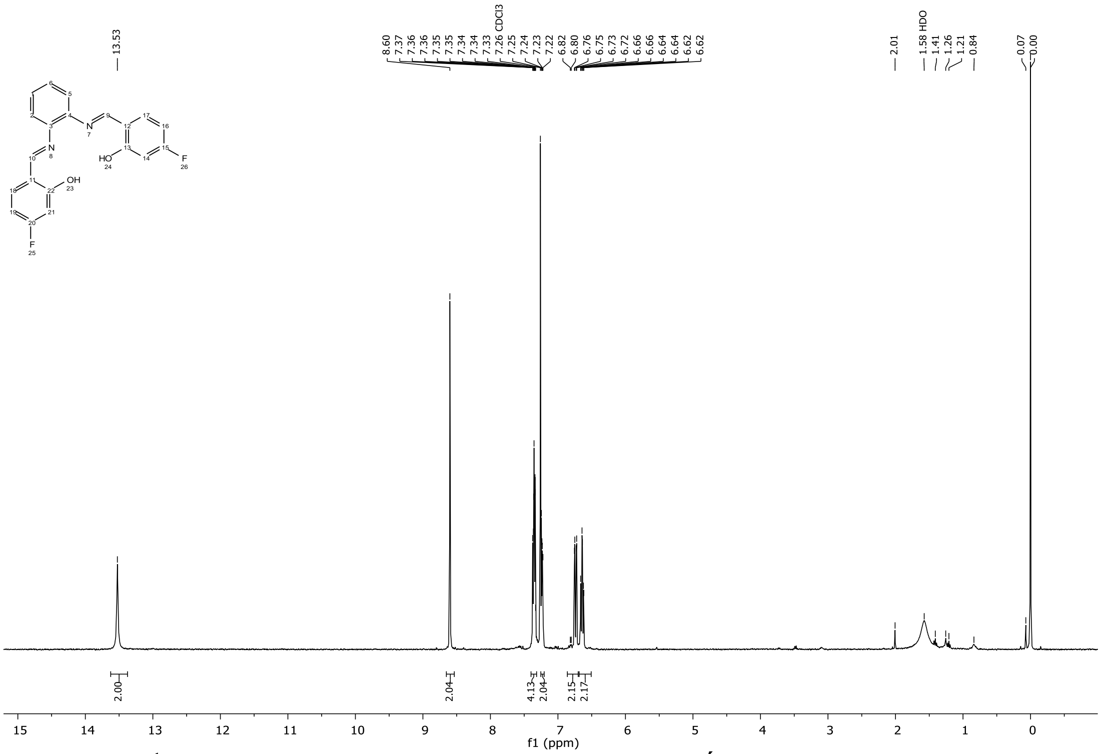

Figure S2: ^{1}H-NMR (400 MHz, chloroform-*d*) of *N,N´*-bis(4-fluorosalicylidene)-1,2-phe-nylenediamine (**L2**)

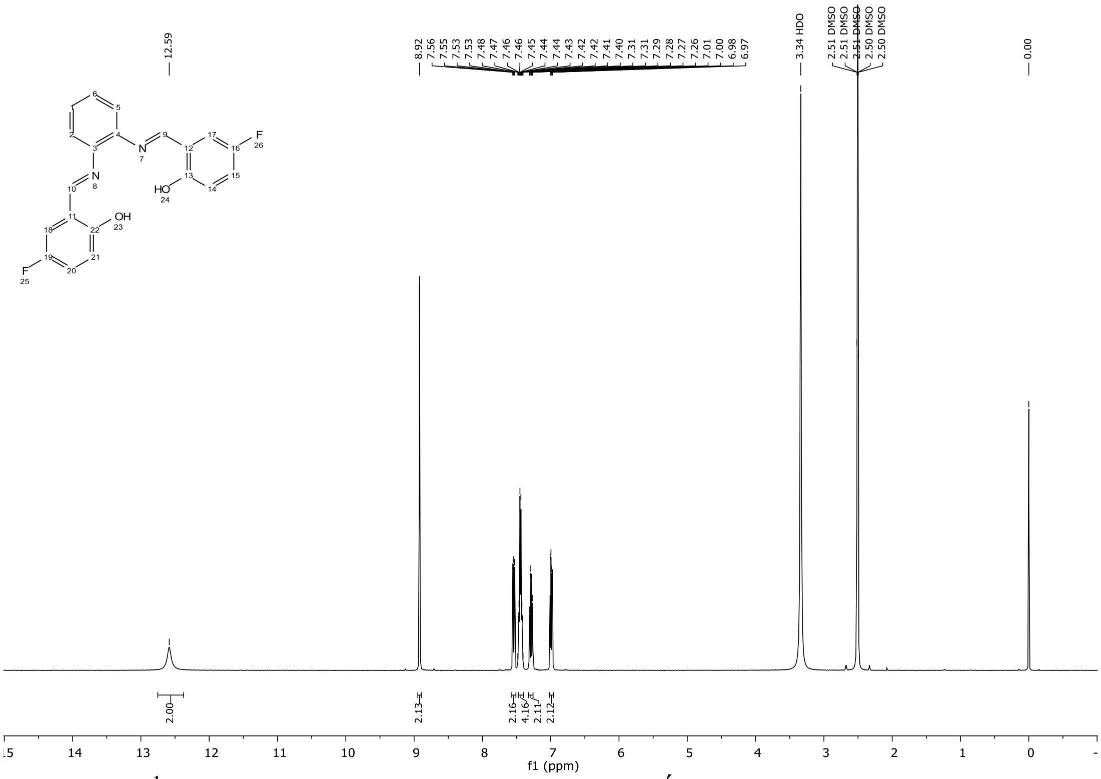

Figure S3: ^{1}H-NMR (400 MHz, DMSO-d_6) of *N,N*′-bis(5-fluorosalicylidene)-1,2-phenylene-diamine (**L3**)

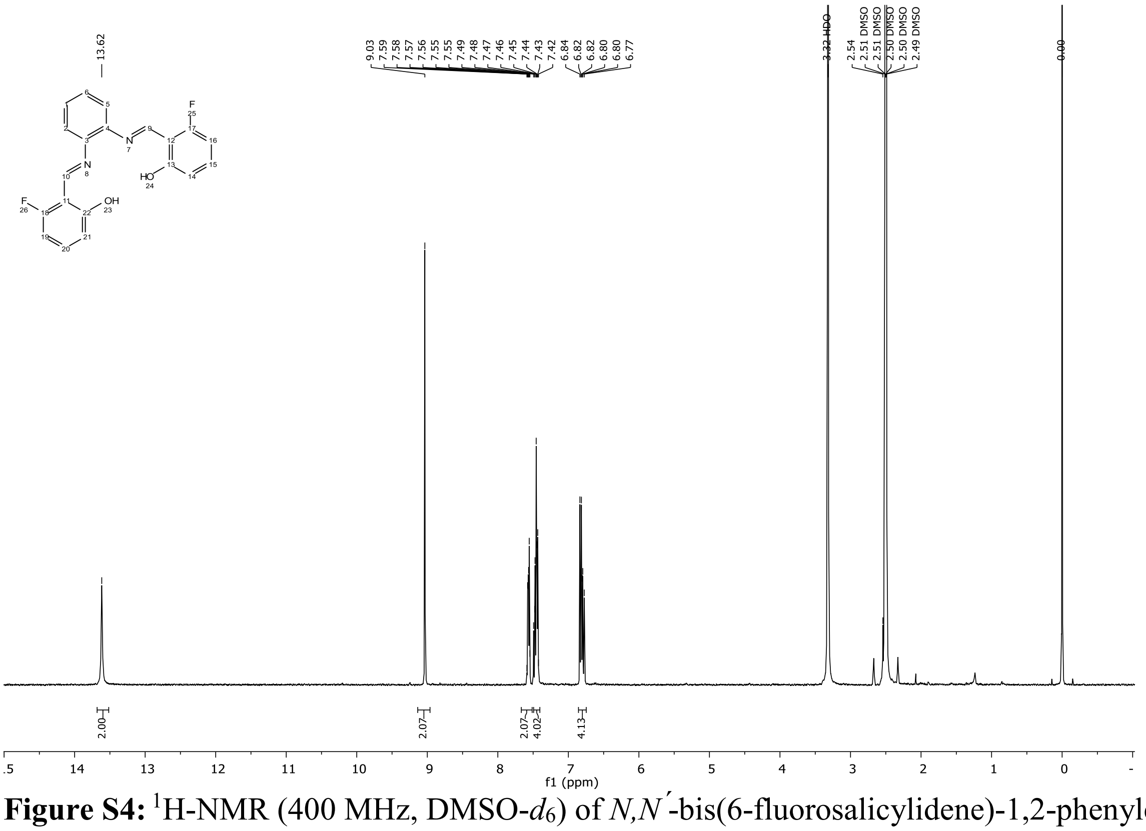

Figure S4: ^{1}H-NMR (400 MHz, DMSO-d_6) of *N,N*′-bis(6-fluorosalicylidene)-1,2-phenylene-diamine (**L4**)

^{13}C-NMR spectra of ligands

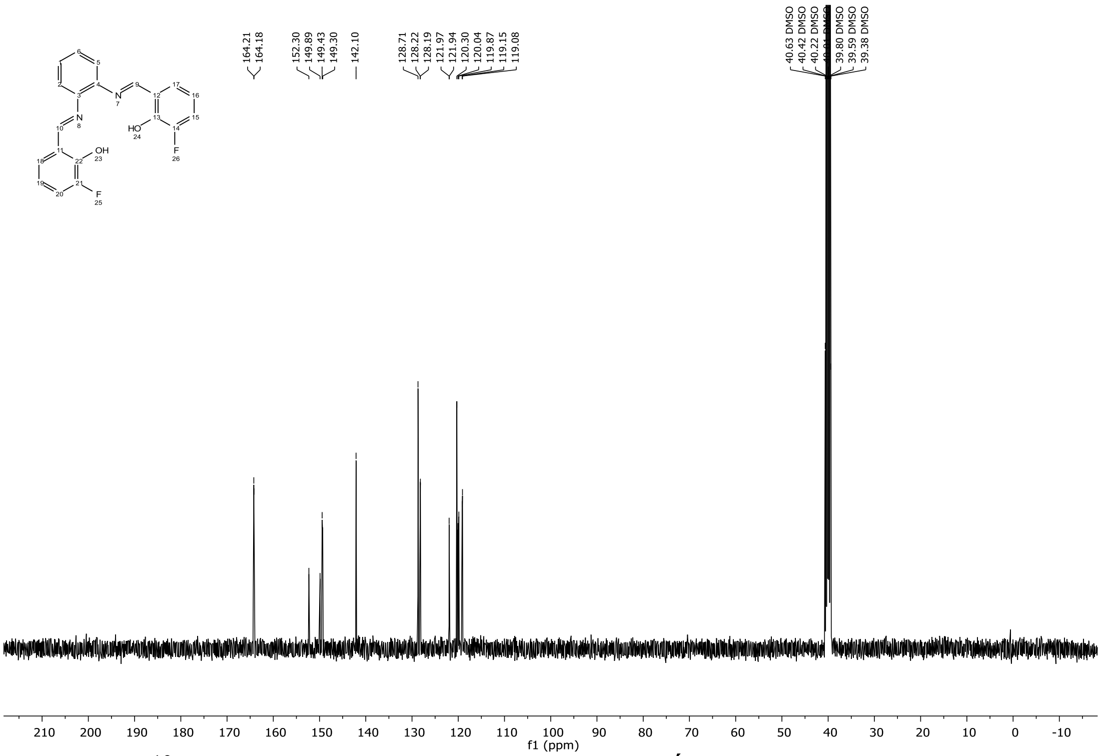

Figure S5: ^{13}C-NMR (101 MHz, DMSO-d_6) of *N,N′*-bis(3-fluorosalicylidene)-1,2-phenylene-diamine (**L1**)

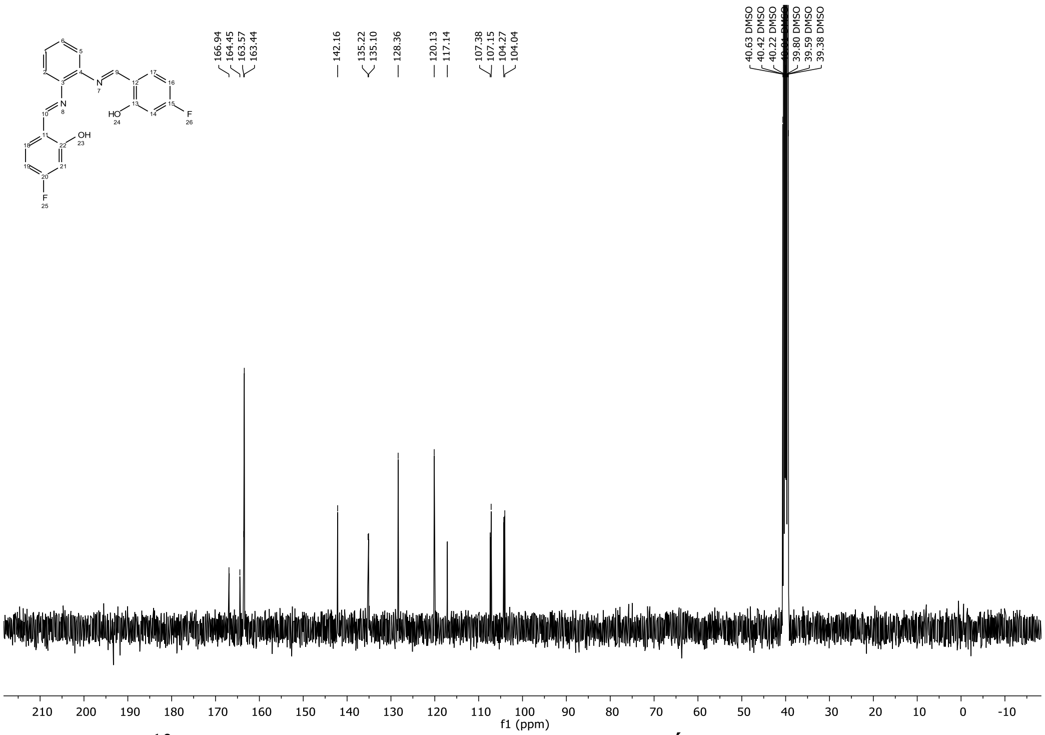

Figure S6: ^{13}C-NMR (101 MHz, DMSO-d_6) of *N,N′*-bis(4-fluorosalicylidene)-1,2-phenylene-diamine (**L2**)

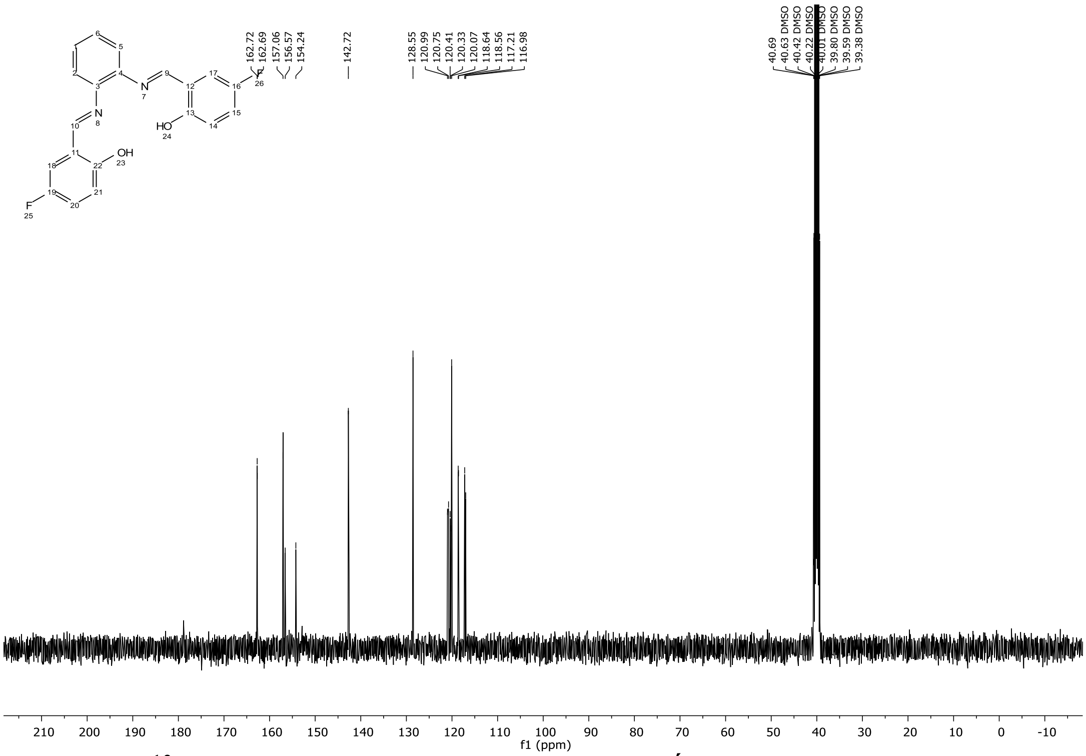

Figure S7: ^{13}C-NMR (101 MHz, DMSO-d_6) of *N,N*′-bis(5-fluorosalicylidene)-1,2-phenylene-diamine (**L3**)

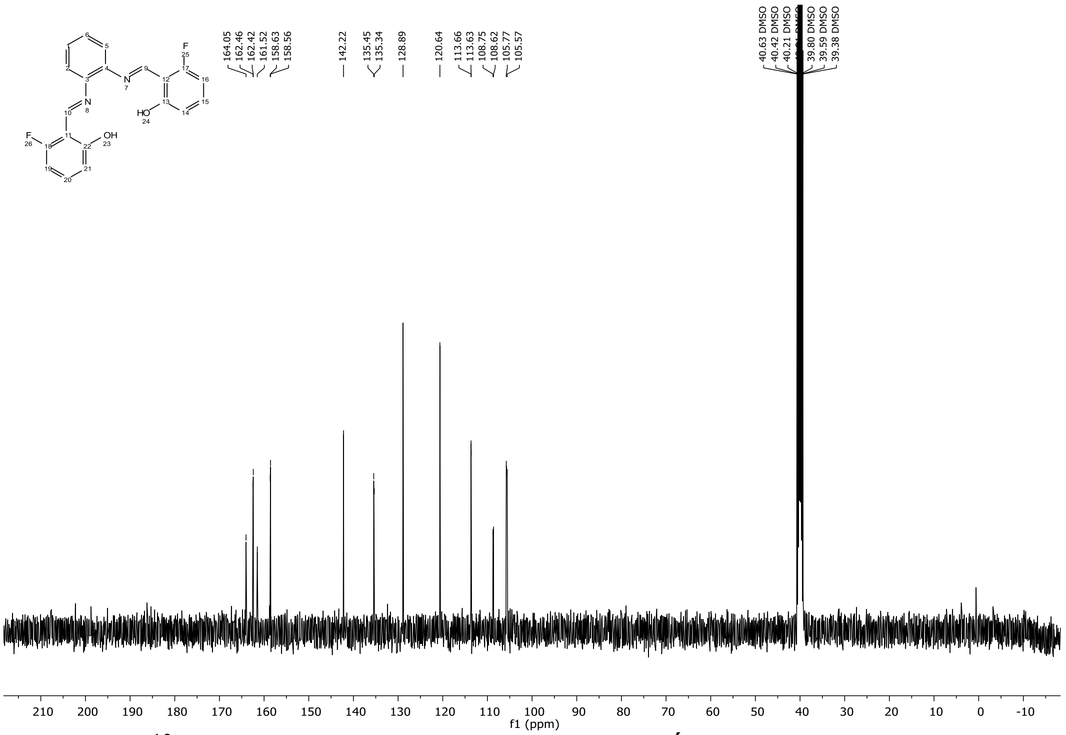

Figure S8: ^{13}C-NMR (101 MHz, DMSO-d_6) of *N,N*′-bis(6-fluorosalicylidene)-1,2-phenylene-diamine (**L4**)

ATR-FTIR spectra of ligands and complexes

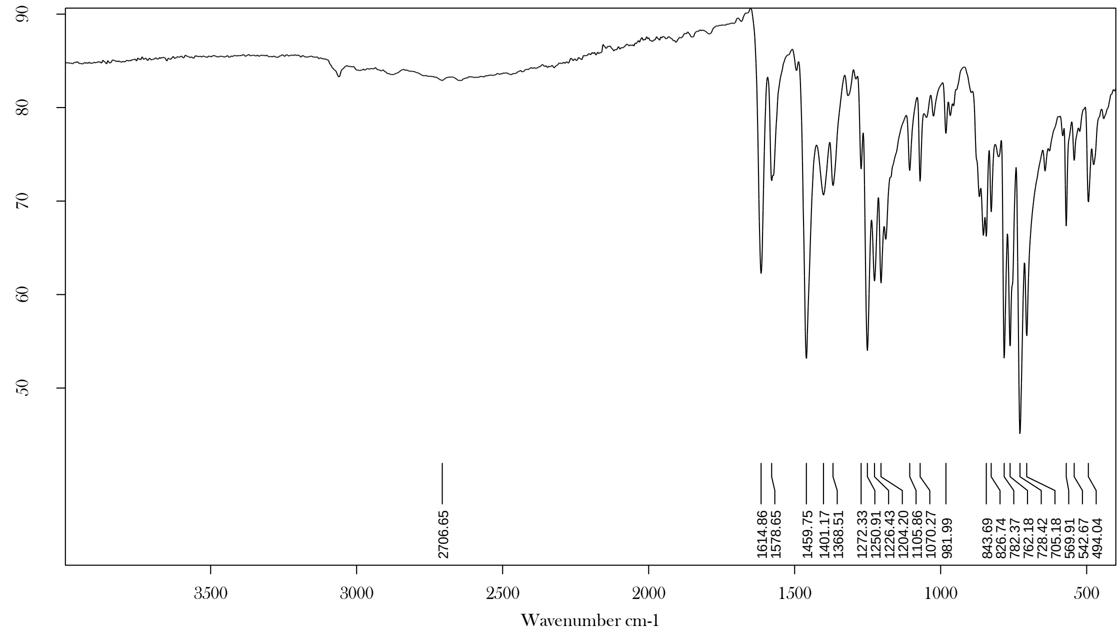

Figure S9: ATR-FTIR spectrum of *N,N´*-bis(3-fluorosalicylidene)-1,2-phenylenediamine (**L1**)

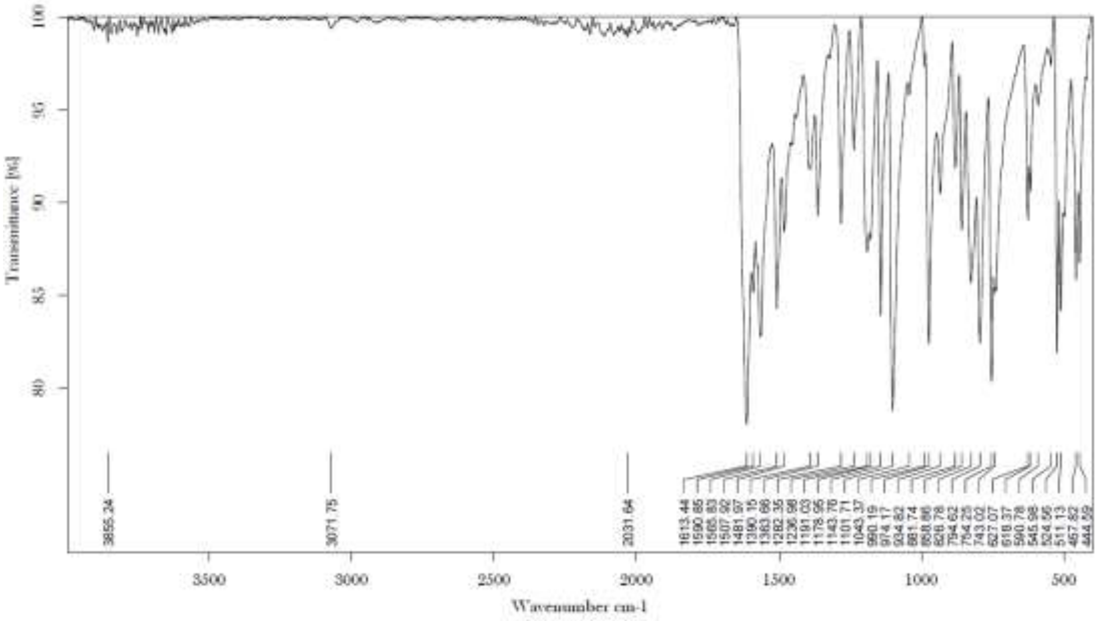

Figure S10: ATR-FTIR spectrum of *N,N´*-bis(4-fluorosalicylidene)-1,2-phenylenediamine (**L2**)

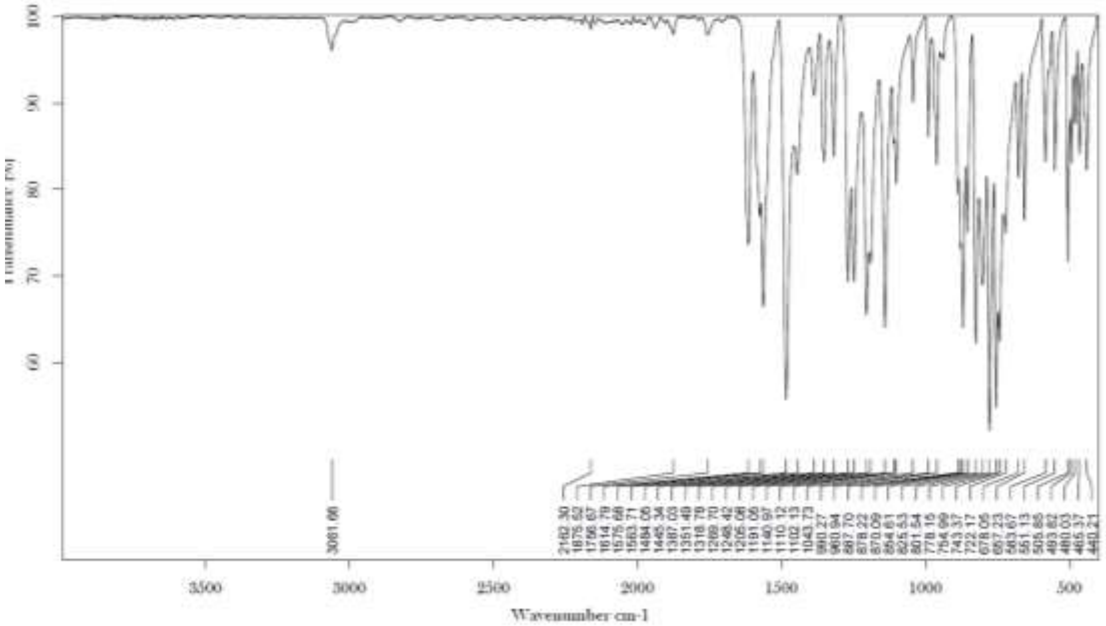

Figure S11: ATR-FTIR spectrum of *N,N*′-bis(5-fluorosalicylidene)-1,2-phenylenediamine (**L3**)

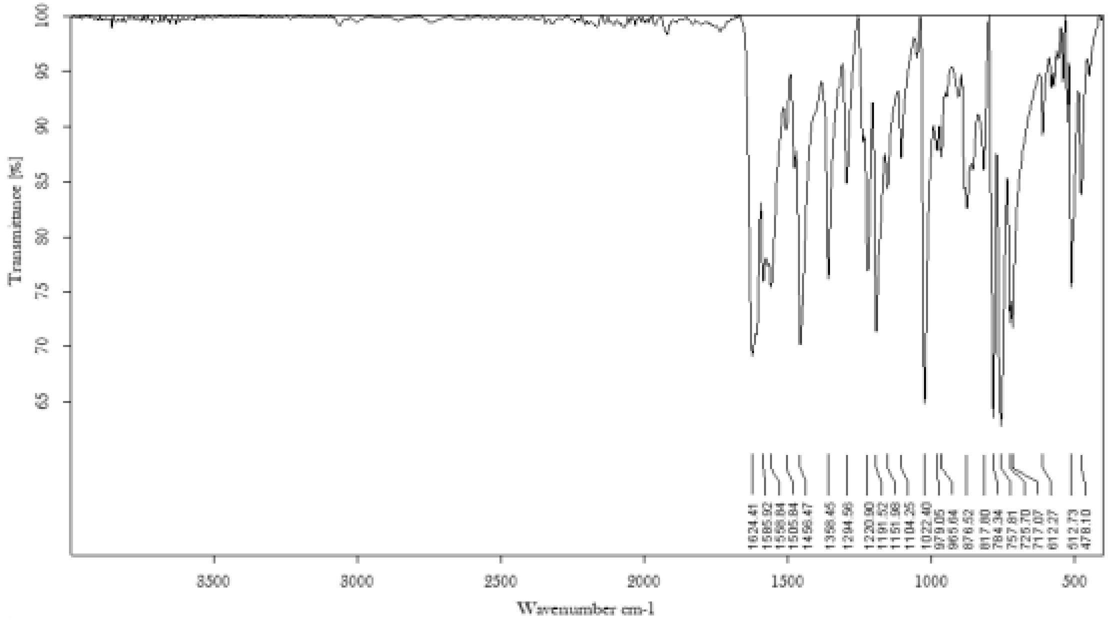

Figure S12: ATR-FTIR spectrum of *N,N*′-bis(6-fluorosalicylidene)-1,2-phenylenediamine (**L4**)

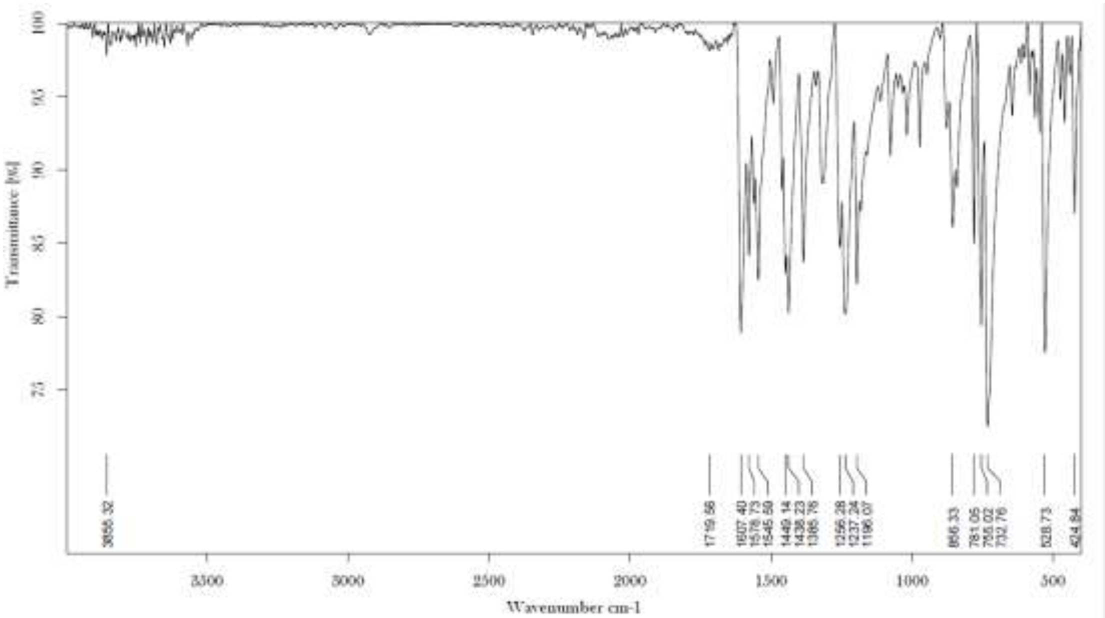

Figure S13: ATR-FTIR spectrum of chlorido[*N,N´*-bis(3-fluorosalicylidene)-1,2-phenylenediamine]iron(III) (**C1**)

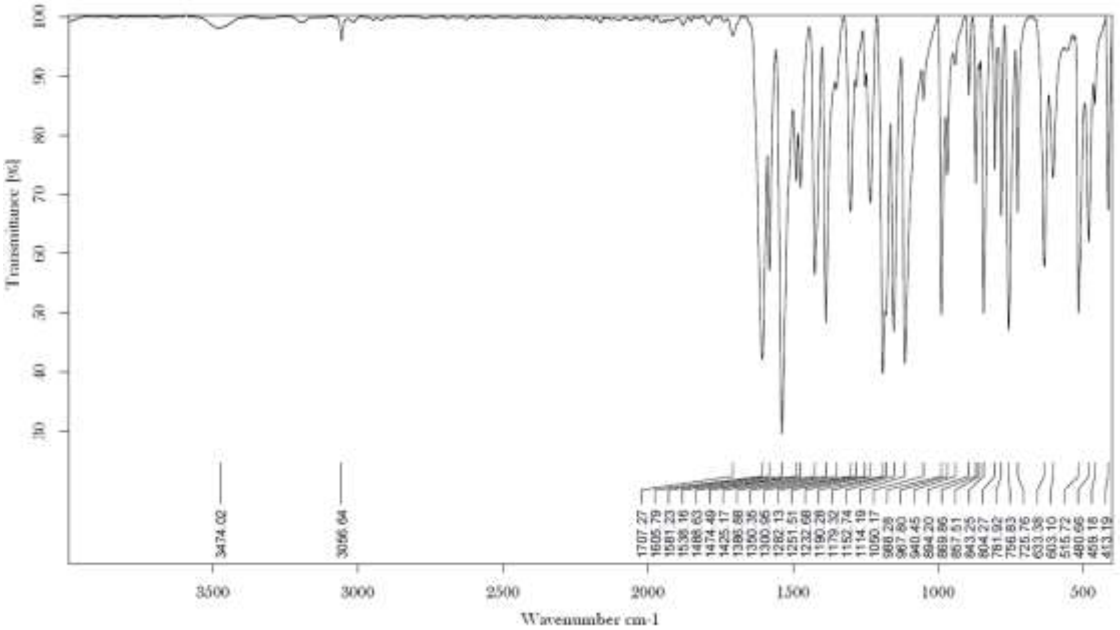

Figure S14: ATR-FTIR spectrum of chlorido[*N,N´*-bis(4-fluorosalicylidene)-1,2-phenylenediamine]iron(III) (**C2**)

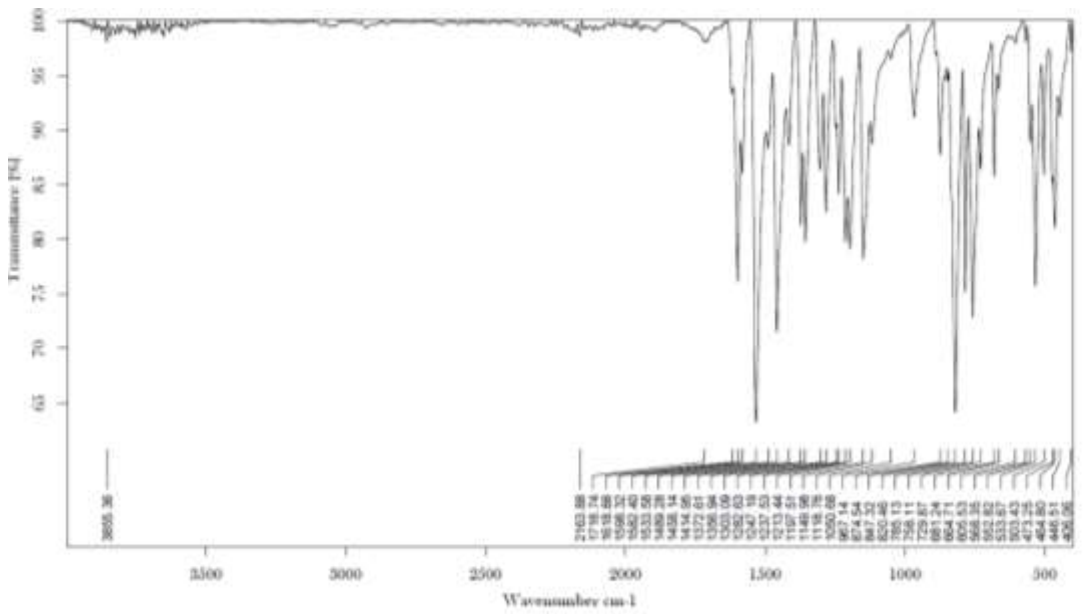

Figure S15: ATR-FTIR spectrum of chlorido[*N,N*´-bis(5-fluorosalicylidene)-1,2-phenylenediamine]iron(III) (**C3**)

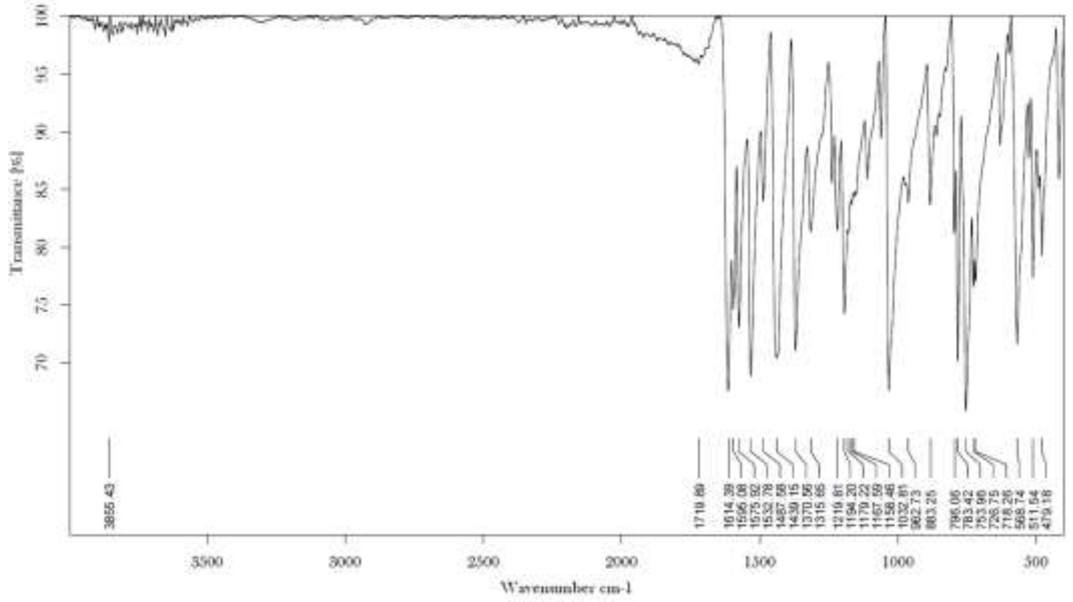

Figure S16: ATR-FTIR spectrum of chlorido[*N,N*´-bis(6-fluorosalicylidene)-1,2-phenylenediamine]iron(III) (**C4**)

Evans ^{1}H-NMR spectra of complexes

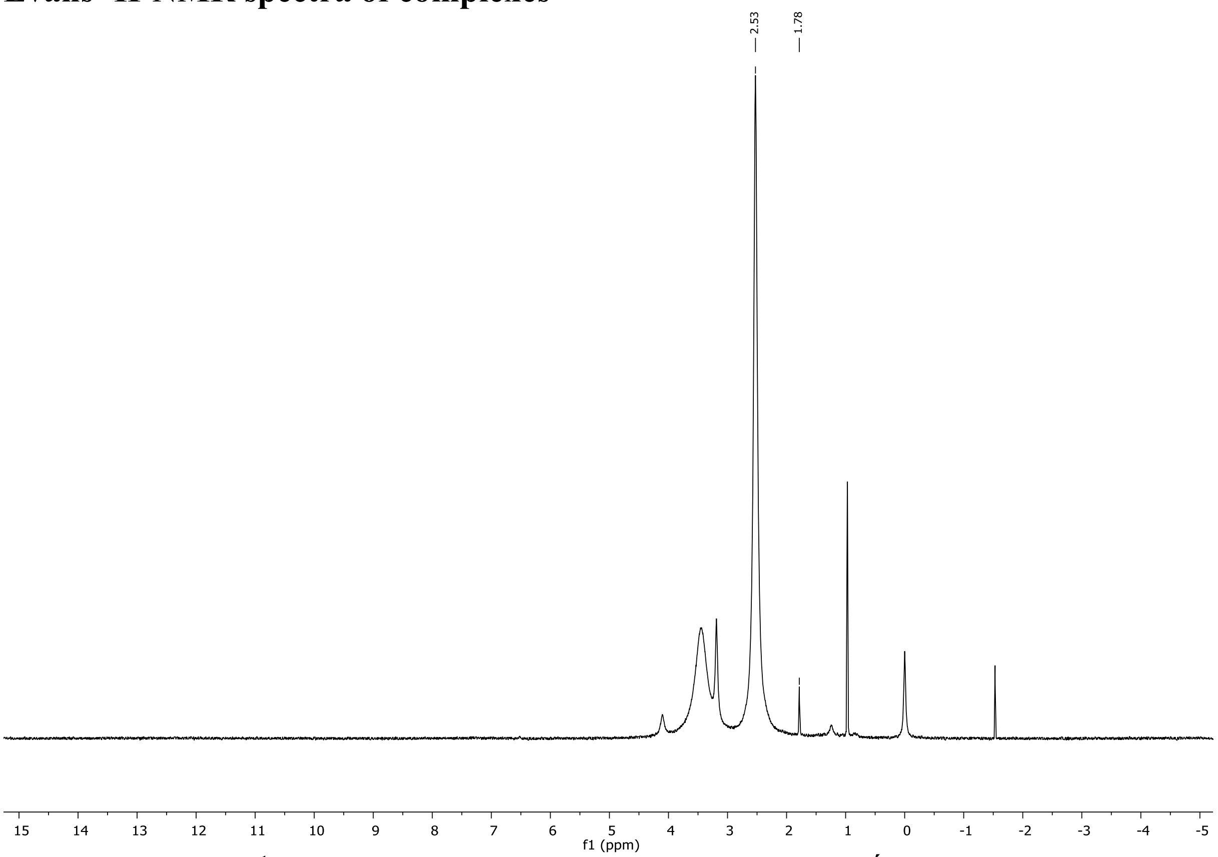

Figure S17: Evans ^{1}H-NMR spectrum (400 MHz) of chlorido[*N,N*′-bis(3-fluorosalicylidene)-1,2-phenylenediamine]iron(III) (**C1**) in DMSO-d_6

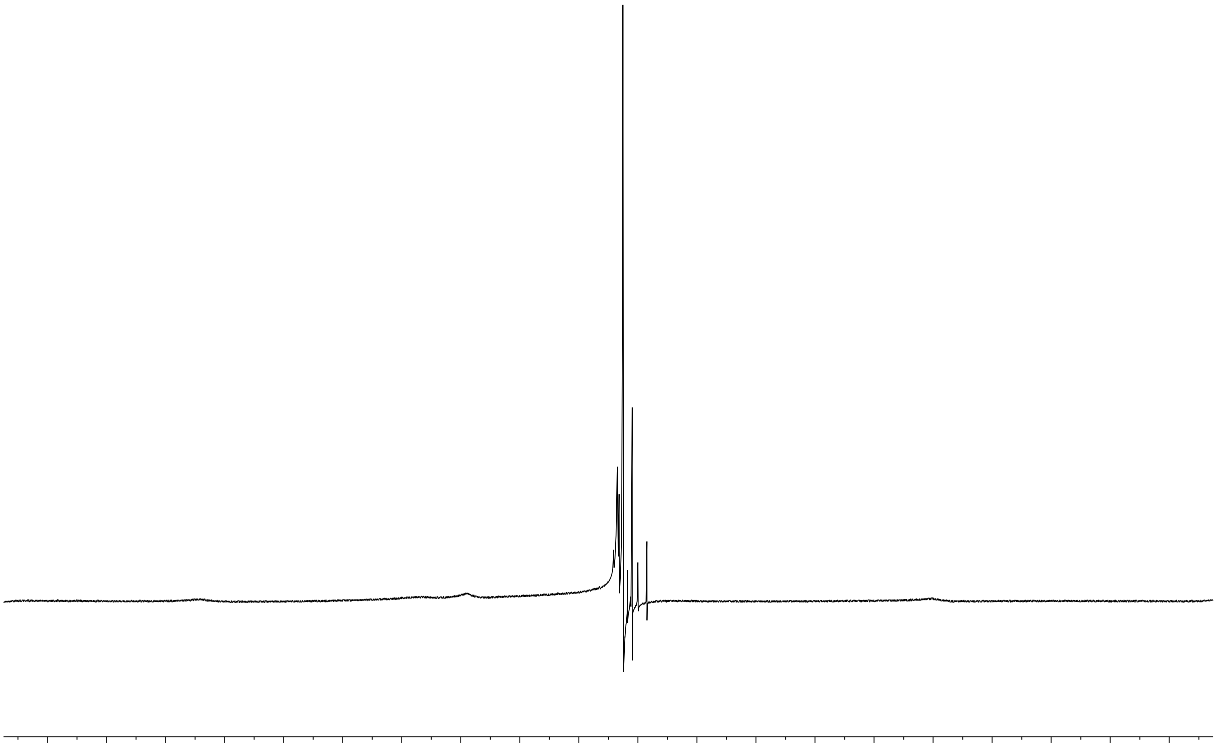

Figure S18: Evans ^{1}H-NMR spectrum (400 MHz) of chlorido[*N,N*′-bis(3-fluorosalicylidene)-1,2-phenylenediamine]iron(III) (**C1**) in DMSO-d_6 between -100 and 100 ppm

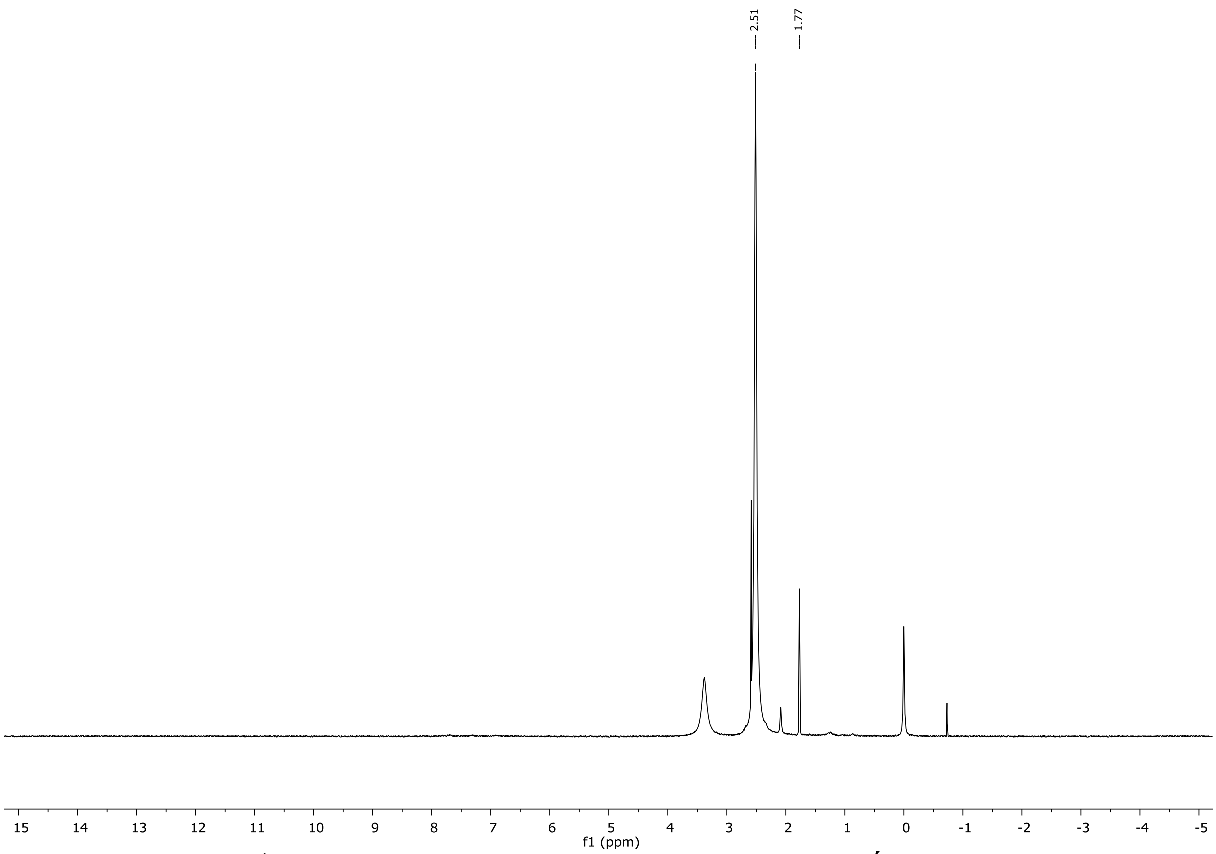

Figure S19: Evans ^{1}H-NMR spectrum (400 MHz) of chlorido[*N,N′*-bis(4-fluorosalicylidene)-1,2-phenylenediamine]iron(III) (**C2**) in DMSO-d_6

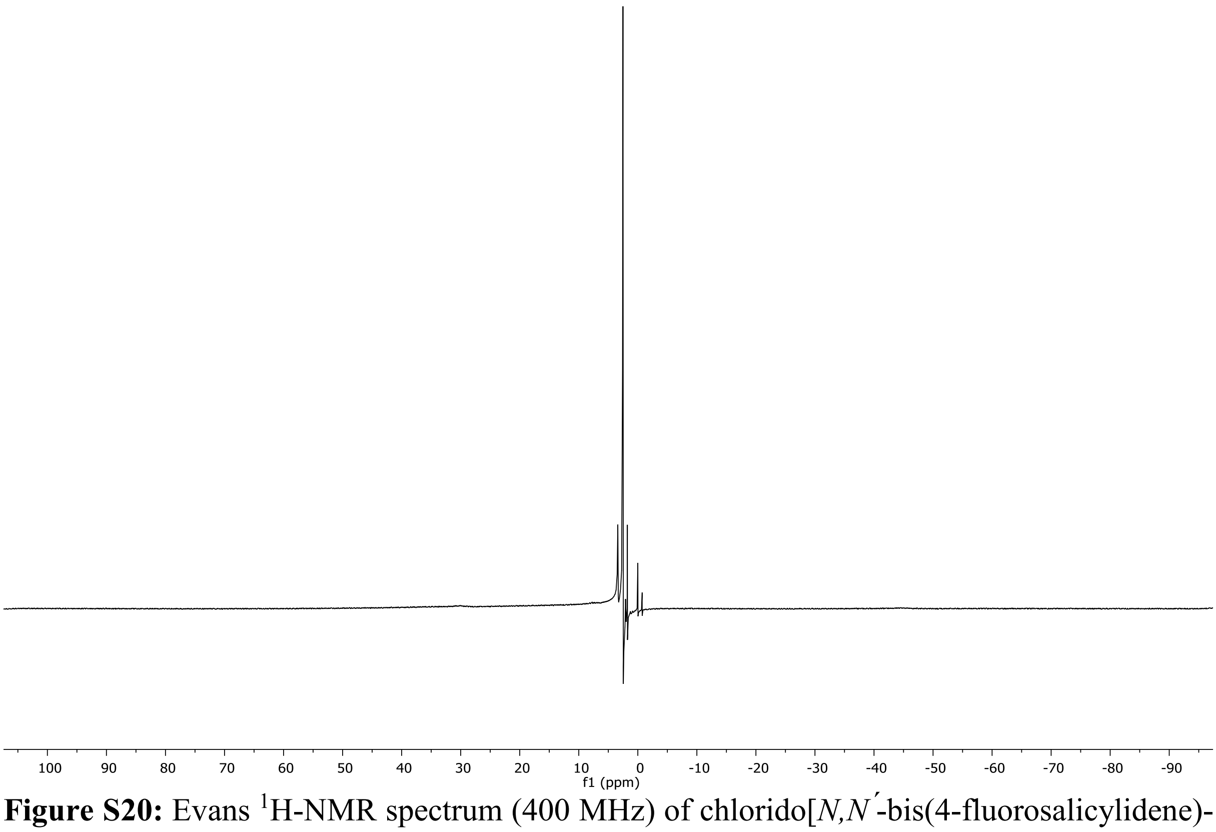

Figure S20: Evans ^{1}H-NMR spectrum (400 MHz) of chlorido[*N,N′*-bis(4-fluorosalicylidene)-1,2-phenylenediamine]iron(III) (**C2**) in DMSO-d_6 between -100 and 100 ppm

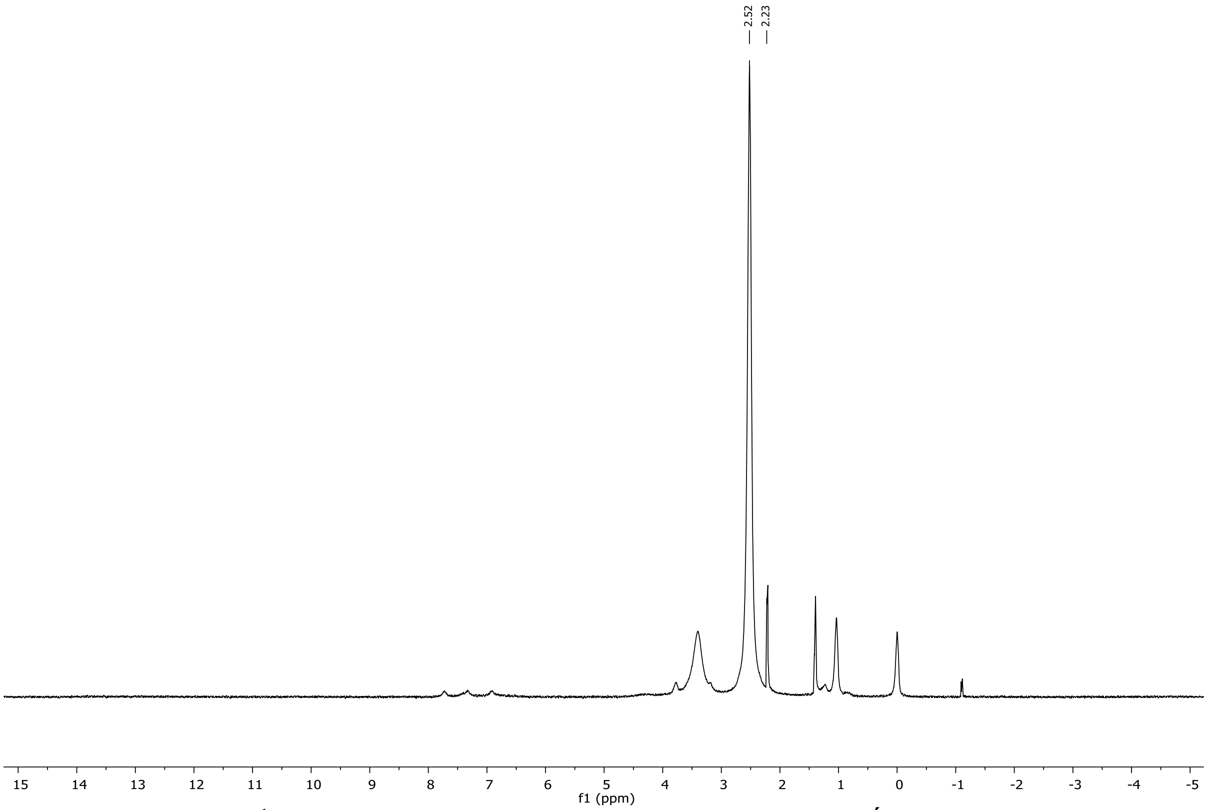

Figure S21: Evans ^{1}H-NMR spectrum (400 MHz) of chlorido[*N,N*′-bis(5-fluorosalicylidene)-1,2-phenylenediamine]iron(III) (**C3**) in DMSO-d_6

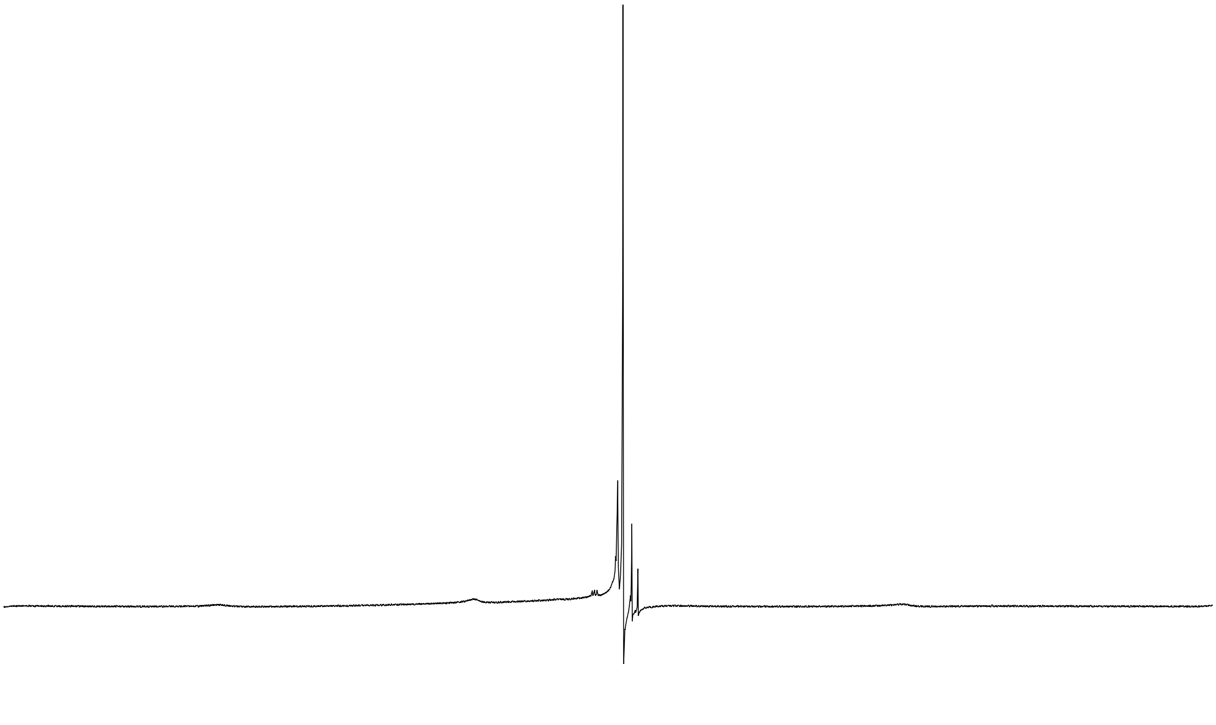

Figure S22: Evans ^{1}H-NMR spectrum (400 MHz) of chlorido[*N,N*′-bis(5-fluorosalicylidene)-1,2-phenylenediamine]iron(III) (**C3**) in DMSO-d_6 between -100 and 100 ppm

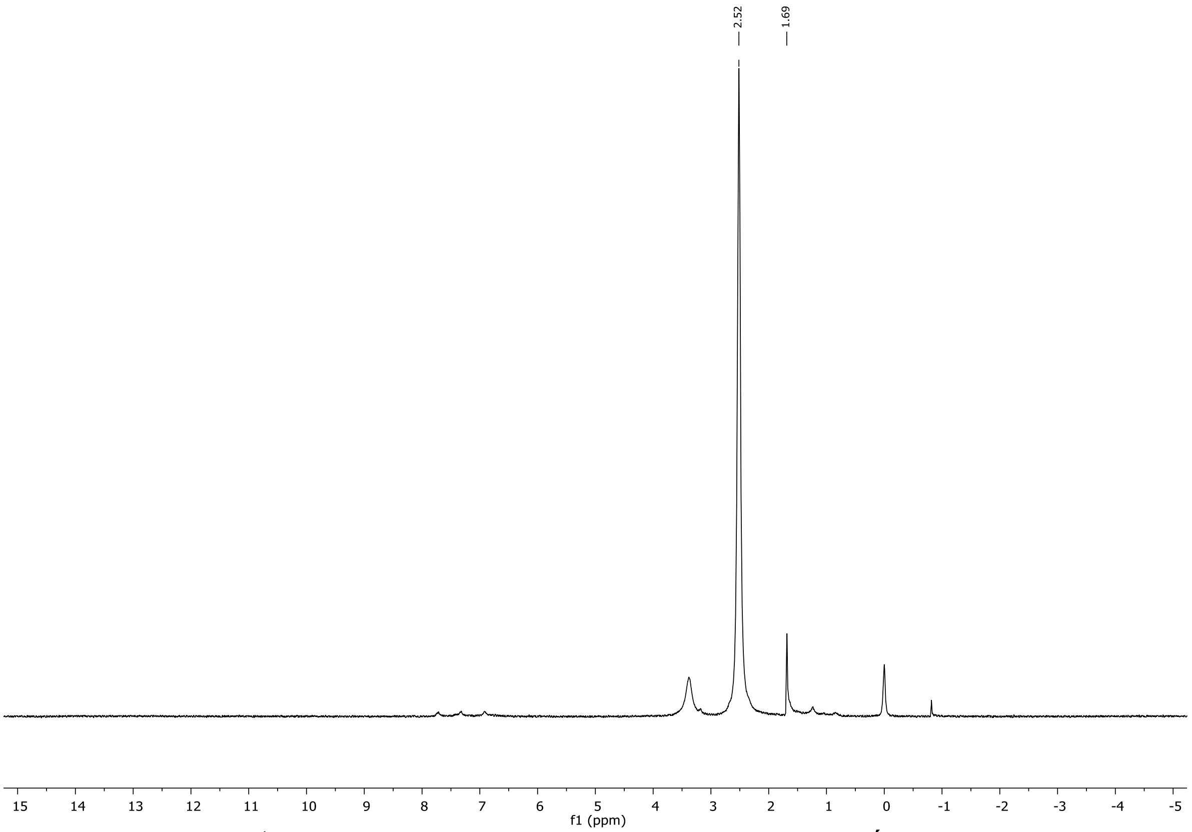

Figure S23: Evans ^{1}H-NMR spectrum (400 MHz) of chlorido[*N,N′*-bis(6-fluorosalicylidene)-1,2-phenylenediamine]iron(III) (**C4**) in DMSO-d_6

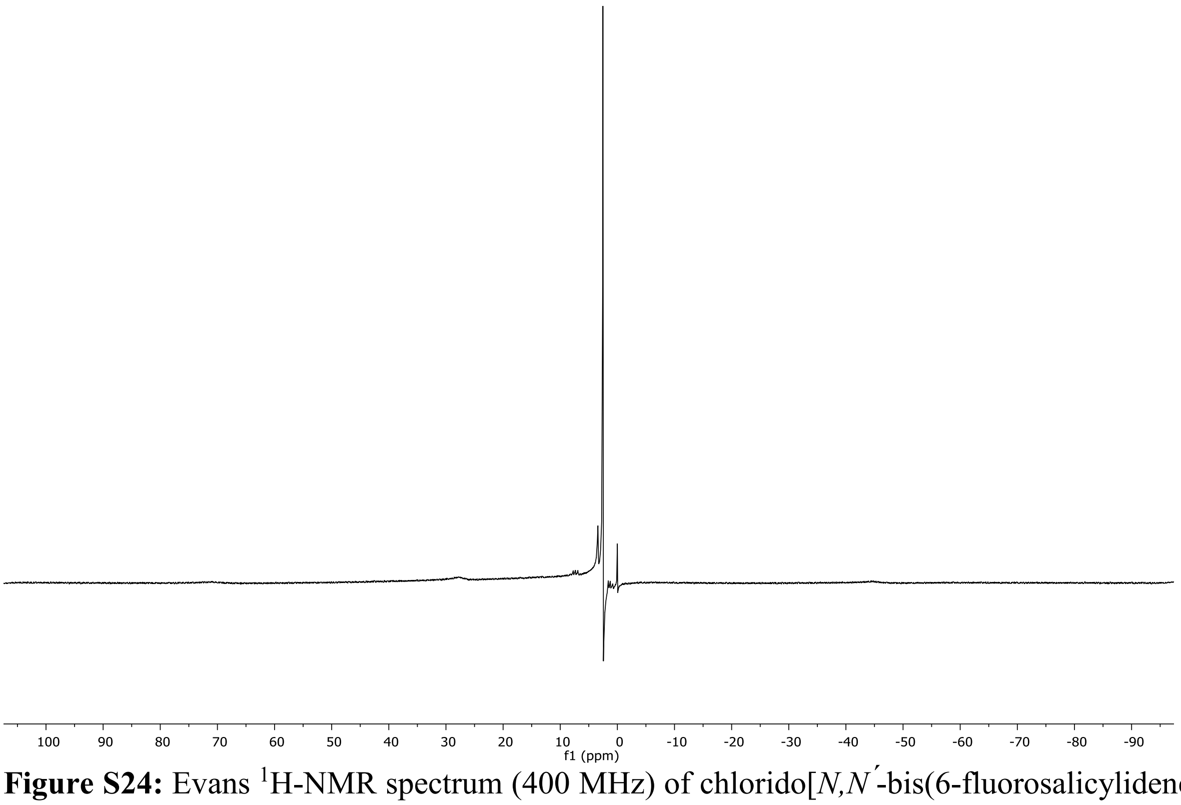

Figure S24: Evans ^{1}H-NMR spectrum (400 MHz) of chlorido[*N,N′*-bis(6-fluorosalicylidene)-1,2-phenylenediamine]iron(III) (**C4**) in DMSO-d_6 between -100 and 100 ppm

EPR spectra of complexes

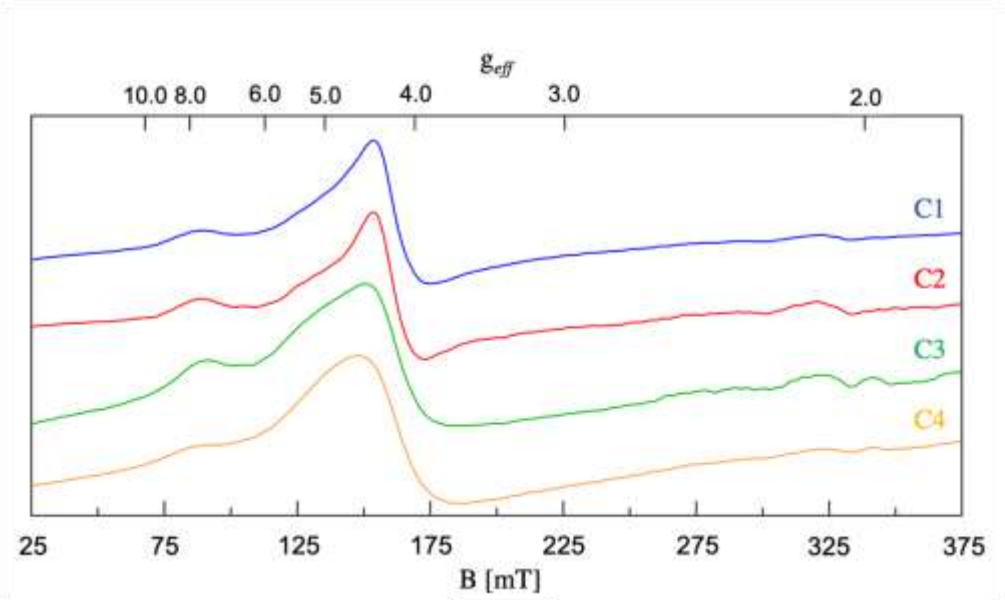

Figure S25: EPR spectrum of **C1** – **C4** in DMSO at 298 K

Cyclic voltammetry of complexes

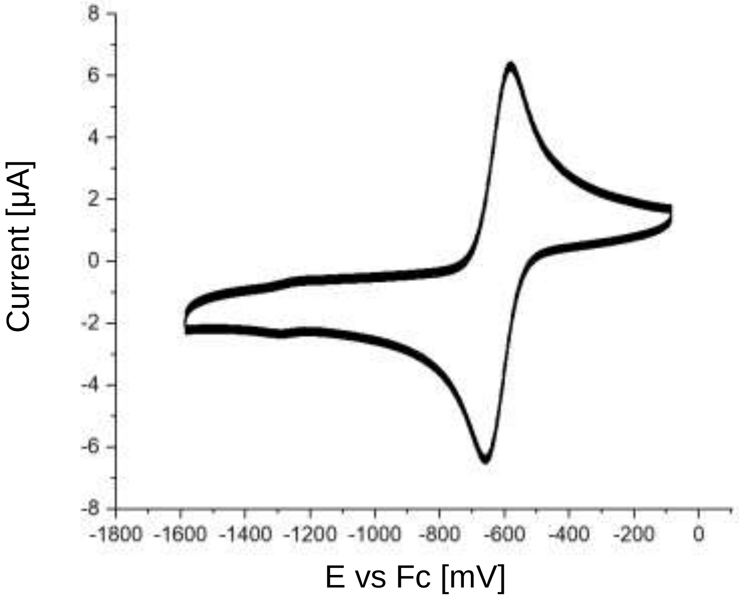

Figure S26: Cyclic voltammogram of **C1** in DMSO

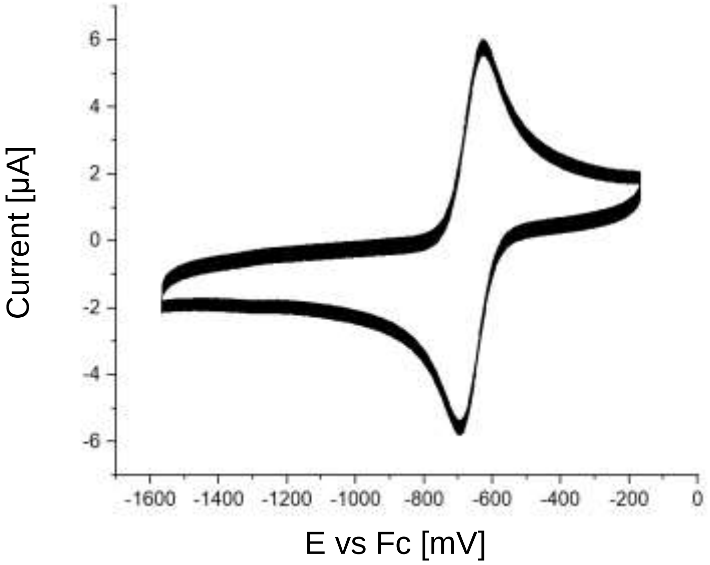

Figure S27: Cyclic voltammogram of **C2** in DMSO

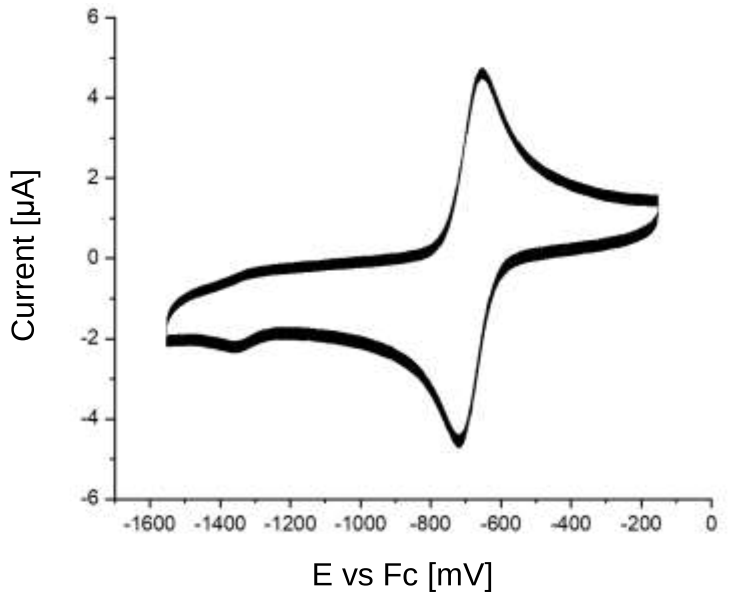

Figure S28: Cyclic voltammogram of **C3** in DMSO

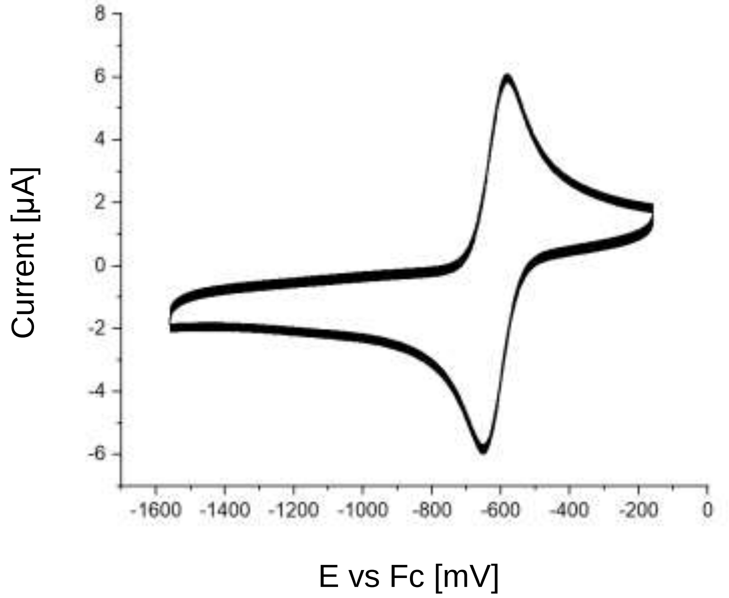

Figure S29: Cyclic voltammogram of **C4** in DMSO

Biological investigations

Determination of the metabolic activity

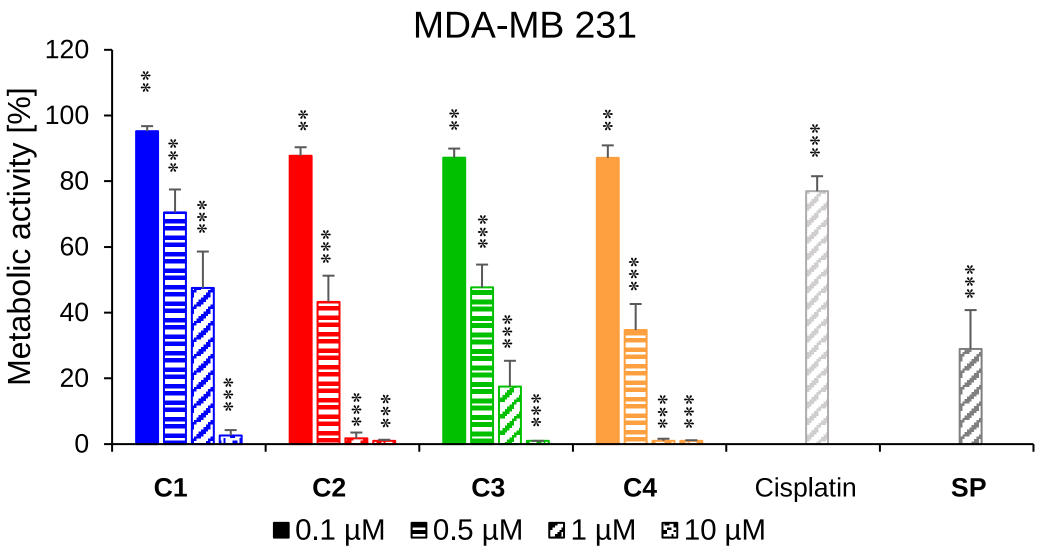

Figure S30: Metabolic activity of MDA-MB 231 cells treated with the complexes **C1** – **C4** at concentrations of 0.1 µM (filled), 0.5 µM (horizontally striped), 1 µM (striped across) and 10 µM (dotted) for 72 h. Cisplatin and **SP** at 1 µM served as references. Metabolic activity in the absence of the complexes was set at 100%. Data are expressed as mean + SE of five independent experiments. The asterisks (** $p<0.005$ and *** $p<0.0005$ against the cells without addition of compound) represent statistical significance.

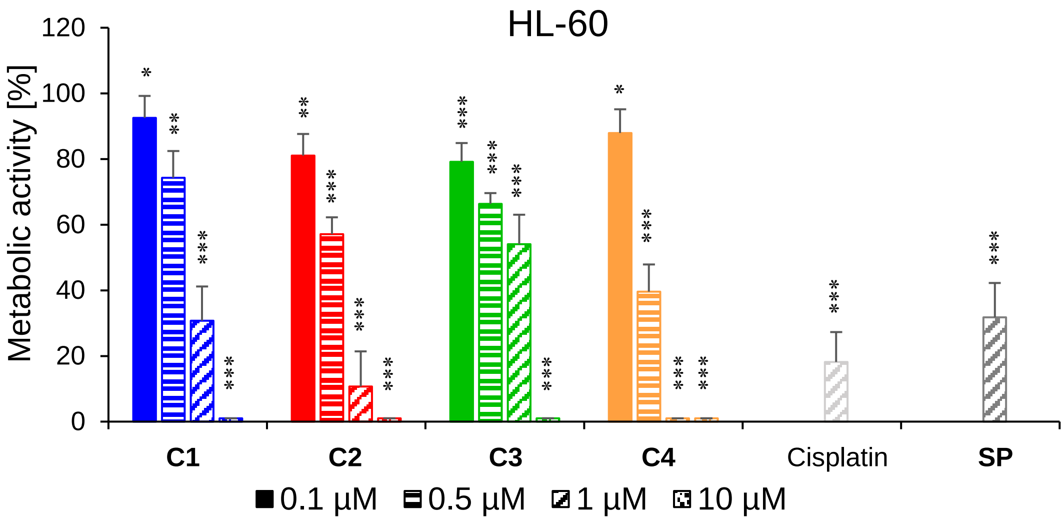

Figure S31: Metabolic activity of HL-60 cells treated with the complexes **C1** – **C4** at concentrations of 0.1 µM (filled), 0.5 µM (horizontally striped), 1 µM (striped across) and 10 µM (dotted) for 72 h. Cisplatin and **SP** at a concentration of 1 µM served as references. Metabolic activity in the absence of the complexes was set at 100%. Data are expressed as mean + SE of five independent experiments. The asterisks (* $p<0.05$, ** $p<0.005$ and *** $p<0.0005$ against the cells without addition of compound) represent statistical significance.

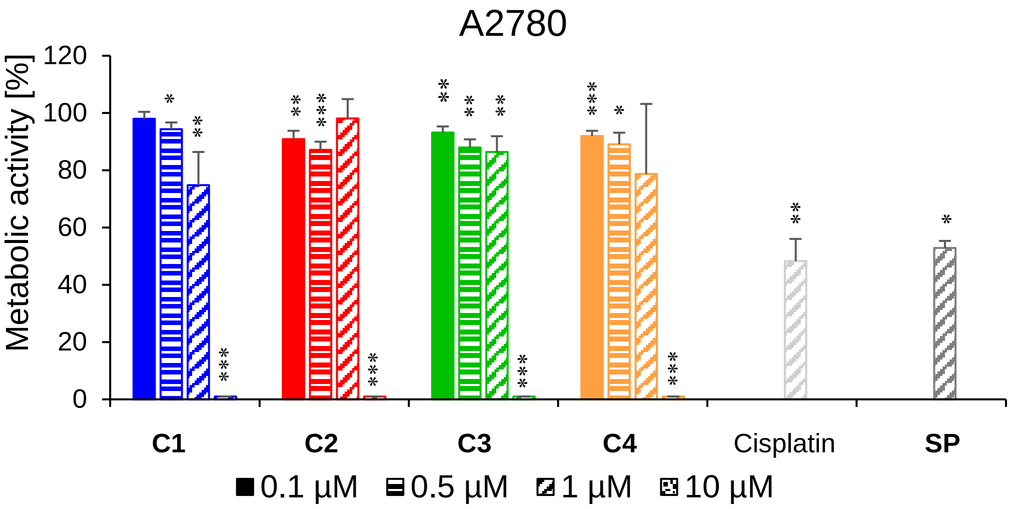

Figure S32: Metabolic activity of A2780 cells treated with the complexes **C1** – **C4** at concentrations of 0.1 µM (filled), 0.5 µM (horizontally striped), 1 µM (striped across) and 10 µM (dotted) for 72 h. Cisplatin and **SP** at a concentration of 1 µM served as references. Metabolic activity in the absence of the complexes was set at 100%. Data are expressed as mean + SE of five independent experiments. The asterisks (* $p<0.05$, ** $p<0.005$ and *** $p<0.0005$ against the cells without addition of compound) represent statistical significance.

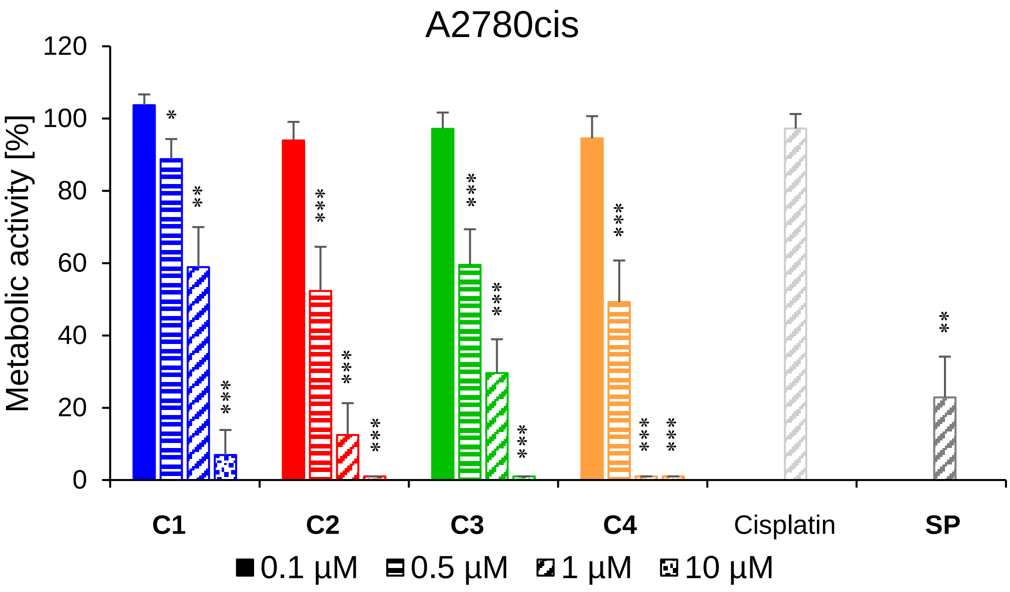

Figure S33: Metabolic activity of A2780cis cells treated with the complexes **C1** – **C4** at concentrations of 0.1 µM (filled), 0.5 µM (horizontally striped), 1 µM (striped across) and 10 µM (dotted) for 72 h. Cisplatin and **SP** at a concentration of 1 µM served as references. Metabolic activity in the absence of the complexes was set at 100%. Data are expressed as mean + SE of five independent experiments. The asterisks (* $p<0.05$, ** $p<0.005$ and *** $p<0.0005$ against the cells without addition of compound) represent statistical significance.

Table S1: IC_{50} values ± SE of complexes **C1** – **C4** determined in MDA-MB 231, HL-60, A2780 and A2780cis cell lines with a modified MTT assay.

Complex	Metabolic Activity IC_{50} [µM]			
	MDA-MB 231	HL-60	A2780	A2780cis
**C1**	0.95 ± 0.58	0.79 ± 0.75	1.43 ± 0.23	1.07 ± 0.79
**C2**	0.50 ± 0.62	0.62 ± 0.40	4.18 ± 0.32	0.54 ± 0.34
**C3**	0.53 ± 0.27	1.61 ± 0.38	2.58 ± 1.79	0.61 ± 0.27
**C4**	0.46 ± 0.60	0.48 ± 0.12	1.79 ± 0.34	0.51 ± 0.13

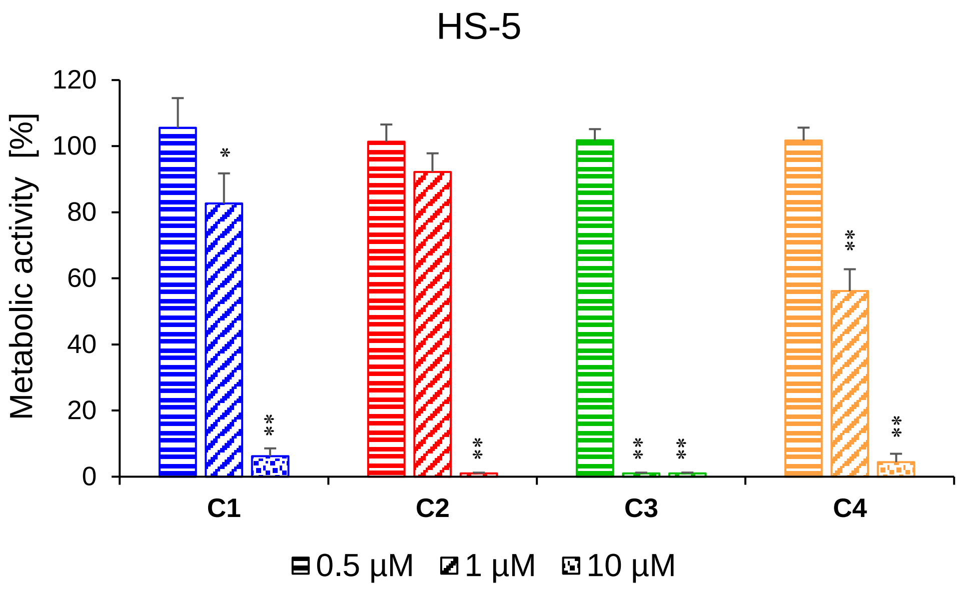

Figure S34: Metabolic activity of HS-5 cells treated with **C1** – **C4** (0.5 µM, 1 µM and 10 µM) for 72 h. Metabolic activity in the absence of the complexes was set at 100%. Data are expressed as mean + SE of six independent experiments. The asterisks (* $p<0.05$ and ** $p<0.005$ against the cells without addition of compound) represent statistical significance.

Fluorescence measurement

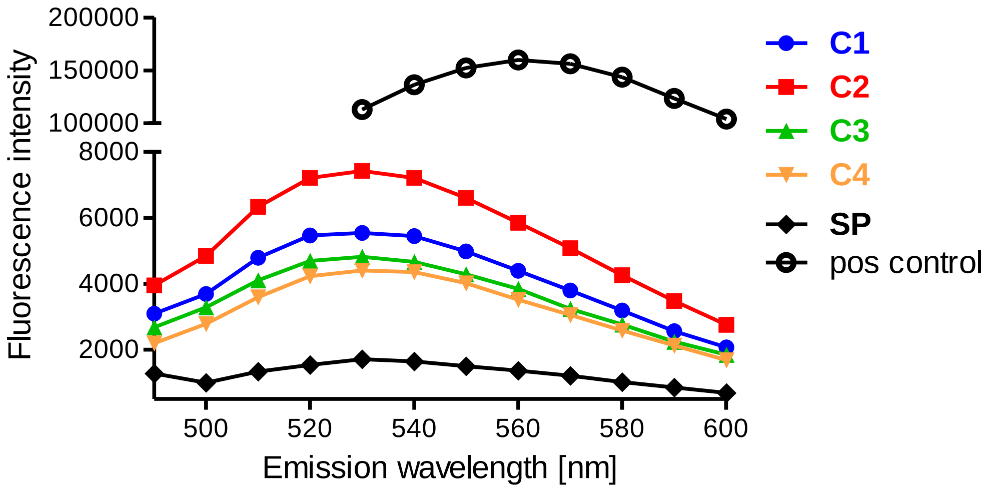

Figure S35: Fluorescence spectra of complexes **C1** – **C4**, **SP** and Coumarin 6 (positive control).

Inverted fluorescence microscopy

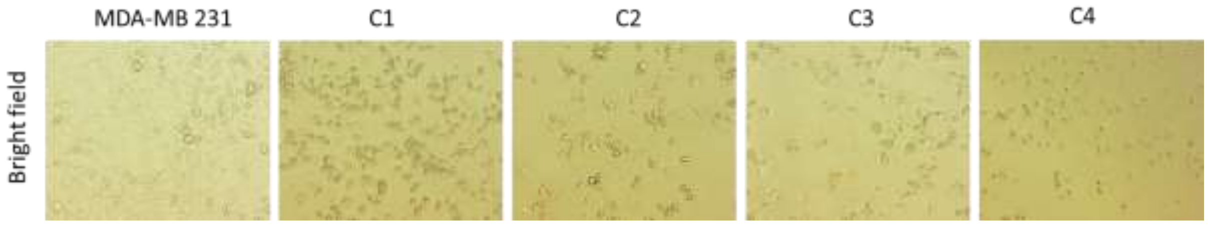

Figure S36: Morphology of MDA-MB 231 cells incubated for 24 h with the complexes **C1** – **C4** at a concentration of 1 µM and, as a control, cells without compound addition, analyzed by an inverted fluorescence microscope.

Western Blot analysis

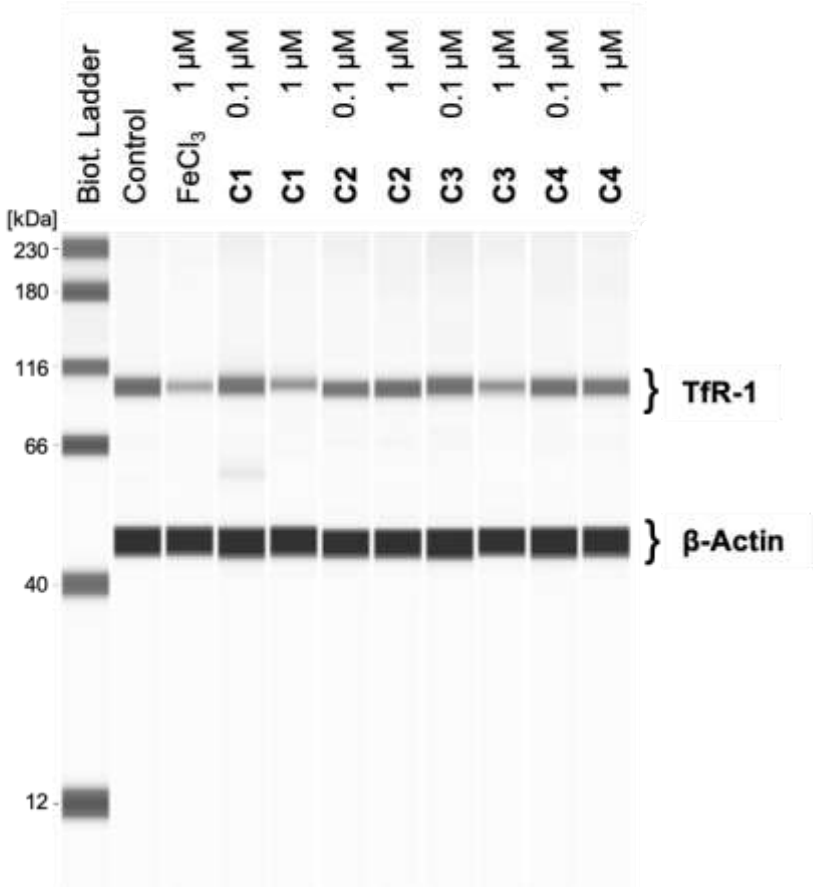

Figure S37: Western Blot analysis of the TfR-1 content in MDA-MB 231 cells in the absence (control; lane 2) and the presence of **C1** – **C4** (0.1 µM and 1 µM, lanes 4-11) after incubation for 24 h. $FeCl_3$ (1 µM; lane 3) served as reference. The biotinylated molecular weight ladder (Biot. Ladder) is shown as lane 1. β-Actin was used as loading control.

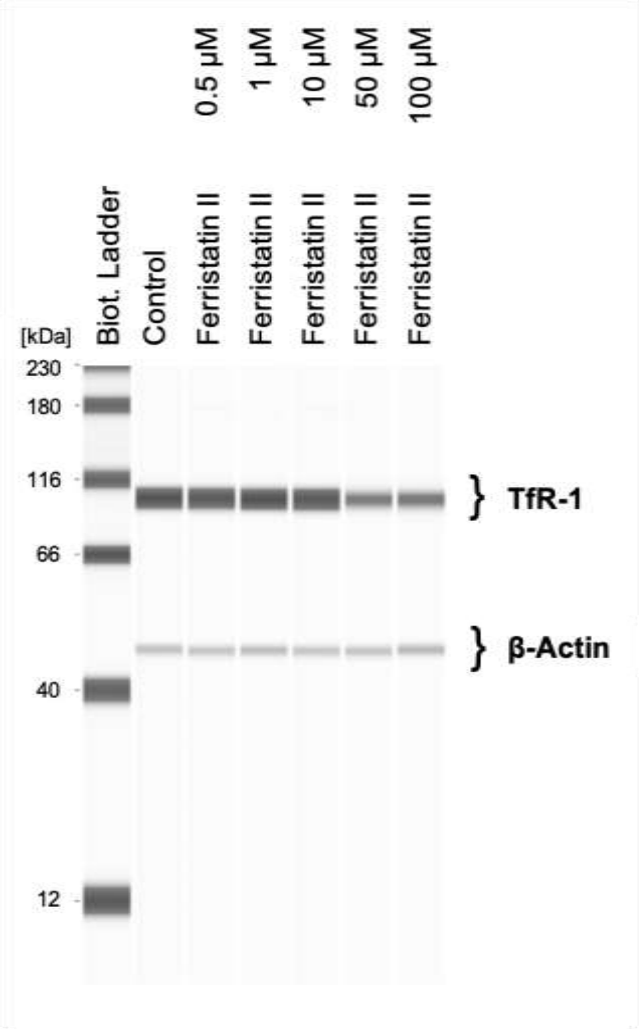

Figure S38: Western Blot analysis of the TfR-1 content in MDA-MB 231 cells in the absence (control; lane 2) and the presence of Ferristatin II at the concentrations of 0.5 µM, 1 µM, 10 µM, 50 µM and 100 µM (lanes 3-7) after incubation for 4 h. The biotinylated molecular weight ladder (Biot. Ladder) is shown as lane 1. β-Actin was used as loading control.

Table S2: Percentage of TfR-1/β-Actin expression in MDA-MB 231 cells after 4 h treatment with Ferristatin II at concentrations ranging from 0.5 µM to 100 µM, respectively. The TfR-1/β-Actin expression of untreated cells was set at 100%.

Ferristatin II Concentration [µM]	TfR-1/β-Actin Expression [%]
0	100.0
0.5	95.0
1	102.4
10	99.5
50	55.2
100	57.5

Flow cytometry

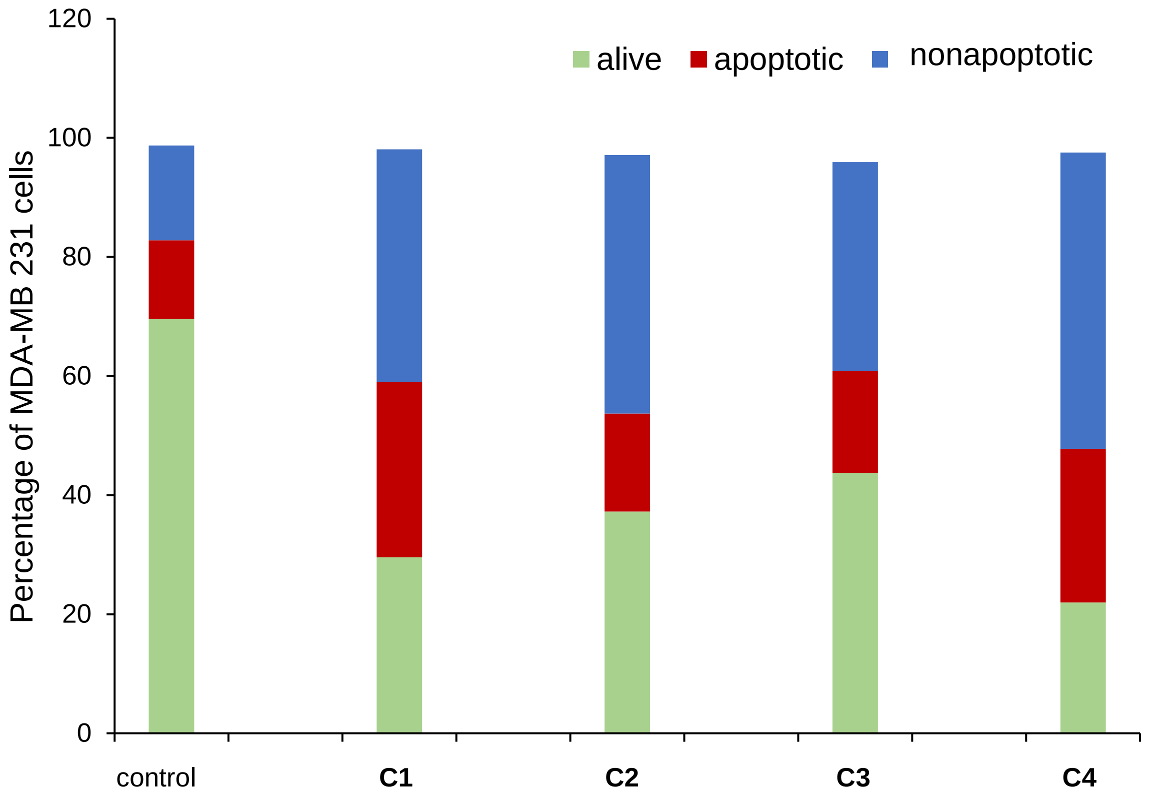

Figure S39: Differentiation of the MDA-MB 231 cell population (%) into alive (green), apoptotic (red) and nonapoptotic (blue) cells after incubation for 24 h without complex (control) and with **C1** – **C4** (1 µM). Data are expressed as mean of two independent experiments.

Determination of the metabolic activity with inhibitors

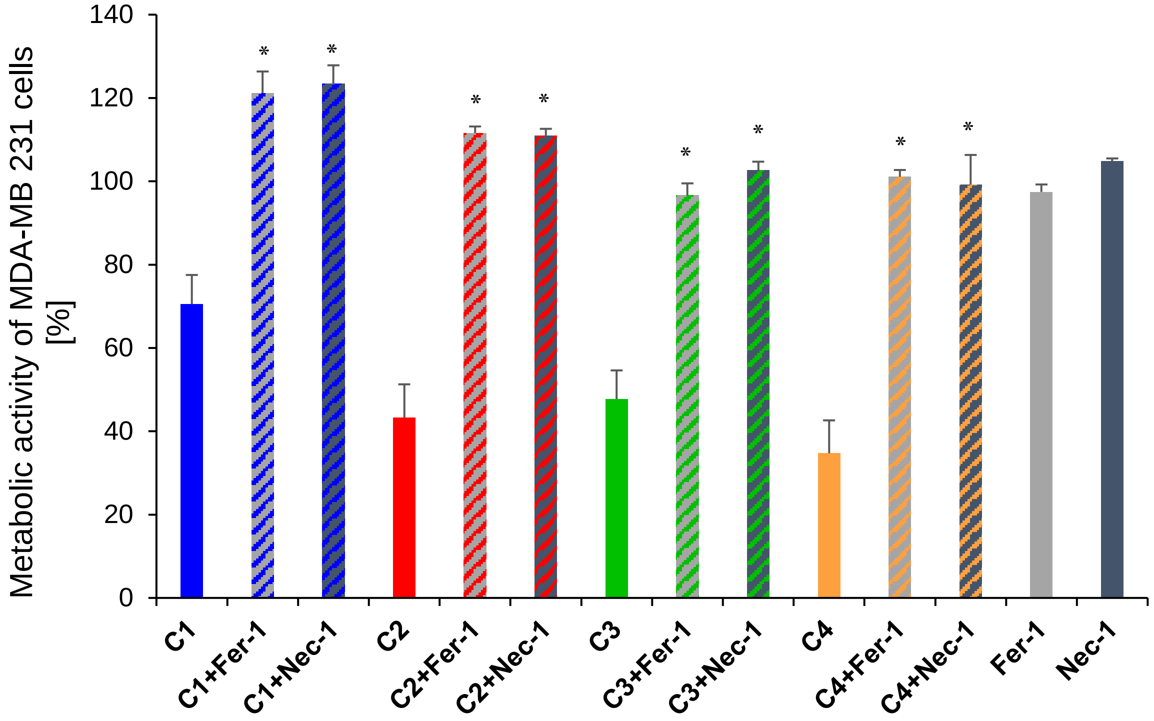

Figure S40: Effect of **C1** – **C4** (0.5 µM) on the metabolic activity of MDA-MB 231 cells in the presence and absence of the ferroptosis inhibitor Fer-1 (1 µM) and the necroptosis inhibitor Nec-1 (20 µM). The inhibitors without compound addition served as references. The mean metabolic activity + SE of four independent experiments is depicted. The metabolic activity of untreated cells was set at 100%. The asterisks (* $p<0.005$ against cells treated with the respective complex without inhibitor) represent statistical significance.

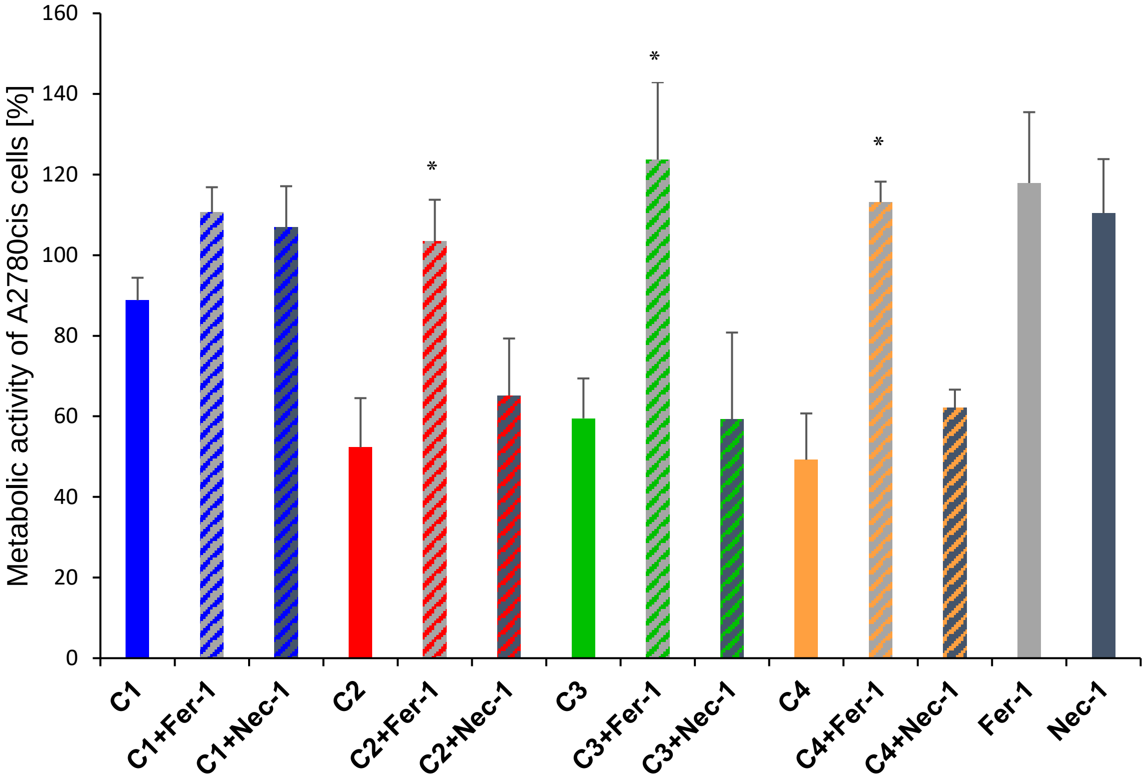

Figure S41: Effect of **C1** – **C4** (0.5 µM) on the metabolic activity of A2780cis cells in the presence and absence of the ferroptosis inhibitor Fer-1 (1 µM) and the necroptosis inhibitor Nec-1 (20 µM). The inhibitors alone served as references. The mean metabolic activity + SE of four independent experiments is depicted. The metabolic activity of untreated cells was set at 100%. The asterisks (* $p<0.05$ against cells treated with the respective complex without inhibitor) represent statistical significance.
